# Loss of Neuron Navigator 2 Impairs Brain and Cerebellar Development

**DOI:** 10.1007/s12311-022-01379-3

**Published:** 2022-02-26

**Authors:** Andrea Accogli, Shenzhao Lu, Ilaria Musante, Paolo Scudieri, Jill A. Rosenfeld, Mariasavina Severino, Simona Baldassari, Michele Iacomino, Antonella Riva, Ganna Balagura, Gianluca Piccolo, Carlo Minetti, Denis Roberto, Fan Xia, Razaali Razak, Emily Lawrence, Mohamed Hussein, Emmanuel Yih-Herng Chang, Michelle Holick, Elisa Calì, Emanuela Aliberto, Rosalba De-Sarro, Antonio Gambardella, Undiagnosed Diseases Network, SYNaPS Study Group, Lisa Emrick, Peter J. A. McCaffery, Margaret Clagett-Dame, Paul C. Marcogliese, Hugo J. Bellen, Seema R. Lalani, Federico Zara, Pasquale Striano, Vincenzo Salpietro

**Affiliations:** 1 Division of Medical Genetics, Department of Specialized Medicine, McGill University, Montreal, Canada; 2 Department of Human Genetics, McGill University, Montreal, QC Canada; 3 Department of Molecular and Human Genetics, Baylor College of Medicine, Houston, TX 77030 USA; 4 Jan and Dan Duncan Neurological Research Institute, Texas Childrens Hospital, Houston, TX 77030 USA; 5 Howard Hughes Medical Institute, Baylor College of Medicine, Houston, TX 77030 USA; 6 Unit of Medical Genetics, IRCCS Istituto Giannina Gaslini, Genoa, Italy; 7 Department of Neurosciences, Rehabilitation, Ophthalmology, Genetics, Maternal and Child Health (DINOGMI), University of Genoa, Genoa, Italy; 8 Neuroradiology Unit, IRCCS Istituto Giannina Gaslini, Genoa, Italy; 9 Pediatric Neurology and Muscular Diseases Unit, IRCCS Giannina Gaslini Institute, Genoa, Italy; 10 Child Neurology and Psychiatry Unit, System Medicine Department, Tor Vergata University of Rome, 00133 Rome, Italy; 11 Baylor Genetics Laboratories, Houston, TX USA; 12 Texas Childrens Hospital, Houston, TX USA; 13 Department of Cardiology, Texas Childrens Hospital, Houston, USA; 14 Department of Ophthalmology, Texas Childrens Hospital, Houston, USA; 15 Retina and Vitreous of Texas, Houston, TX USA; 16 Department of Pediatrics, Division of Neurology and Developmental Neuroscience, Baylor College of Medicine, Houston, TX USA; 17 Department of Neuromuscular Diseases, University College London, Queen Square Institute of Neurology, London, WC1N 3BG UK; 18 Casa Di Cura La Madonnina, via Quadronno 29, 20122 Milano, Italy; 19 Department of Clinical and Experimental Medicine, Policlinic “G. Martino”, University of Messina, 98100 Messina, Italy; 20 Department of Medical and Surgical Sciences, Universita’ Degli Studi “Magna Graecia” Viale Europa, 88100 CATANZARO, Italy; 21 Institute of Medical Sciences, University of Aberdeen, Foresterhill, Aberdeen, UK; 22 Department of Biochemistry, College of Agricultural and Life Sciences, University of Wisconsin, Madison, WI 53706 USA; 23 Pharmaceutical Sciences Division, School of Pharmacy, University of Wisconsin, Madison, WI 53706 USA

**Keywords:** NAV2, Cerebellar hypoplasia, Cerebellar cortical dysplasia, Neuron migration, Axon elongation, Brain malformation

## Abstract

Cerebellar hypoplasia and dysplasia encompass a group of clinically and genetically heterogeneous disorders frequently associated with neurodevelopmental impairment. The Neuron Navigator 2 (*NAV2*) gene (MIM: 607,026) encodes a member of the Neuron Navigator protein family, widely expressed within the central nervous system (CNS), and particularly abundant in the developing cerebellum. Evidence across different species supports a pivotal function of NAV2 in cytoskeletal dynamics and neurite outgrowth. Specifically, deficiency of *Nav2* in mice leads to cerebellar hypoplasia with abnormal foliation due to impaired axonal outgrowth. However, little is known about the involvement of the *NAV2* gene in human disease phenotypes. In this study, we identified a female affected with neurodevelopmental impairment and a complex brain and cardiac malformations in which clinical exome sequencing led to the identification of *NAV2* biallelic truncating variants. Through protein expression analysis and cell migration assay in patient-derived fibroblasts, we provide evidence linking NAV2 deficiency to cellular migration deficits. In model organisms, the overall CNS histopathology of the *Nav2* hypomorphic mouse revealed developmental anomalies including cerebellar hypoplasia and dysplasia, corpus callosum hypo-dysgenesis, and agenesis of the olfactory bulbs. Lastly, we show that the *NAV2* ortholog in *Drosophila*, *sickie* (*sick*) is widely expressed in the fly brain, and *sick* mutants are mostly lethal with surviving escapers showing neurobehavioral phenotypes. In summary, our results unveil a novel human neurodevelopmental disorder due to genetic loss of *NAV2*, highlighting a critical conserved role of the *NAV2* gene in brain and cerebellar development across species.

**Supplementary Information:**

The online version contains supplementary material available at 10.1007/s12311-022-01379-3.

## Introduction

In humans, development of the cerebellum begins around the ninth week of gestation and continues postnatally following highly orchestrated processes that involve a series of complex morphogenic events [1]. These events are tightly regulated by intra- and extra-cellular molecular pathways, which promote and regulate neuronal proliferation, differentiation, and migration, resulting in the formation of a foliated and lobulated structure that plays a key role in motor and cognitive functions [2]. Acquired or genetic disruptions that impair the complex regulatory machinery of cerebellar development may result in a broad array of diverse congenital anomalies frequently associated with neurodevelopmental disorders (NDDs) [3]. Among these, cerebellar vermis hypoplasia is the most common, often representing a non-specific finding in a large proportion of individuals affected with intellectual disability [4]. In contrast, cerebellar dysplasia is very rare and is often part of a more complex brain malformation [5]. Despite the significant advances in our understanding of the molecular basis of cerebellar malformations, roughly half of individuals with cerebellar hypoplasia remain genetically undiagnosed [6].

The *NAV2* gene (MIM: 607,026) encodes the Neuron Navigator 2 protein, a member of the Neuron Navigator protein family that is abundantly expressed in the developing central nervous system (CNS) [7, 8] and known to affect cytoskeletal dynamics [9]. *NAV2* was first identified as an all-*trans* retinoic acid responsive gene in human neuroblastoma SH-SY5Y cell line [10, 11], having pivotal functions in neurite outgrowth and axon elongation [9]. Homologs to *NAV2* are present in animal models such as *Drosophila* (*sick*) and *Caenorhabditis elegans* (*unc-53*) and are well known to regulate cell migration, neurite outgrowth, and axon elongation [12, 13]. Notably, transgene expression of the human full-length *NAV2* was able to rescue the mechanosensory neuron axon elongation defects in the *unc-53* mutant [9]. The critical role of NAV2 in neuronal migration and axon elongation has also been observed in the hypomorphic *Nav2* mutant mice that display ataxia due to defects in the development of cerebellar vermis, characterized by reduced cerebellar granule cell migration and impaired axonal outgrowth [14]. Despite robust evidence underscoring the importance of NAV2 in CNS development and function in both vertebrate and invertebrate models, no patients have been reported with mutations in *NAV2*. To explore whether Neuron Navigator 2 affects human brain and cerebellum development and other phenotypes, we screened (whole or clinical) exome sequencing (ES) data from individuals affected with (sporadic) molecularly undetermined cerebellar dysplasia for de novo or biallelic variants in the *NAV2* gene. This led to the identification of compound heterozygous truncating variants in *NAV2* in a female individual affected with developmental delay and a complex brain malformation including vermian hypoplasia and cerebellar cortical dysplasia. Cellular studies in this patient fibroblasts showed decreased and aberrant *NAV2* transcripts and proteins. Cell migration assays on patient cells link NAV2 deficiency to perturbed migration processes. The comparison of *Nav2* hypomorphic mouse histopathology and patient neuroimaging features revealed strikingly overlapping brain and cerebellar findings in humans and mice. Moreover, the *NAV2* ortholog in flies, *sick*, is required for proper mushroom body development as well as proper motor and neurobehavioral functions, thus unveiling a critical role of NAV2 for brain development across different species.

## Materials and Methods

### Discovery Cohort

We screened for biallelic and/or de novo variants in *NAV2* genomic datasets part of the GIGA (Gaslini IIT Genomic Alliance) and the SYNaPS study group consortia (which are involved in the genetic investigation of rare undiagnosed pediatric neurodevelopmental disorders) that include ES data of about 30,000 families; furthermore, we interrogated publicly available databases, including DECIPHER (https://www.deciphergenomics.org/), LOVD (https://www.lovd.nl/), and the Matchmaker Exchange platform GeneMatcher[15]. Written informed consent for patients who underwent ES was obtained under protocols approved by local institutional review boards. We only included cases with detailed clinical phenotyping and available brain imaging. We also excluded cases with a prior genetic diagnosis or an established candidate disease-causing variant. No de novo variants were identified in *NAV2* and a fully segregating biallelic variant was found in a patient that was genetically investigated at Baylor Genetics Laboratories and previously submitted to GeneMatcher. In this patient, no pathogenic or candidate variants in any of the known disease genes were found, and clinical trio ES led to the identification of compound heterozygous variants that should cause early truncations in the *NAV2* transcripts/proteins that were initially classified of uncertain significance. Since this gene was not associated with human disease, the family was subsequently enrolled in the Undiagnosed Diseases Network (UDN) to further explore the molecular etiology of her symptoms.

### Exome Sequencing and Variant Analysis

Clinical ES was performed on DNA isolated from peripheral blood of affected patients and their parents when available, as previously described [16, 17]. Sequencing data were processed using commercial tools for the execution of the GATK Best Practices pipeline for ES variant analysis. Exon-level read counts, removal of duplicate reads, mean coverage of coding sequence regions, alignment, and variant annotation were performed using analytical pipelines that include publicly available tools and custom scripts. We looked at non synonymous-exonic and splicing variants with a minor allele frequency ≤ 0.001 in gnomAD database. Validation, parental origin of the resulting variants, and family segregation studies were performed by Sanger sequencing. Variants were interpreted according to the ACMG criteria.

### Histopathological Analysis of Mutant Nav2 Mice

To assess the role of NAV2 in both brain and cerebellar development, we conducted a detailed re-evaluation of the histopathological features of the *Nav2* hypomorphic mutant mice that were previously published [14]. For this purpose, we analyzed cerebellum as well as all other brain regions in Nissl-stained Sects. (30 μm) from two litter matched pairs of wild-type and hypomorphic *Nav2* mice at 8 weeks of age that had been backcrossed 20 times into a C57BL/6 background. All animal studies were performed under an approved IACUC animal protocol according to institutional guidelines at the University of Wisconsin-Madison.

### Western Blot Analysis

The fibroblast cell lysates were prepared using lysis buffer (50 mM Tris–Cl pH 7.4, 150 mM NaCl, 1 mM EDTA, 1% Triton X-100, and 5% β-mercaptoethanol) supplemented with a protease inhibitor cocktail (Roche). Protein concentration of each sample was determined using the Bradford method (Bio-Rad). In total, 40 µg of total protein was denatured at 95 °C and separated on 4–15% Mini-PROTEAN gel (Bio-Rad). The gel was transferred on to a nitrocellulose membrane using Trans-Blot Turbo Transfer System and Trans-Blot Turbo Mini 0.2 µm Nitrocellulose Transfer Pack (Bio-Rad). NAV2 protein was detected using NAV2 Polyclonal Antibody (1:1000, PA5103968—ThermoFisher Scientific) and rabbit secondary antibody (1:10,000, Millipore). Clarity™ Western ECL Substrate (Bio-Rad) was used for the detection of the signals. Image was acquired by Uvitec Mini HD9 (Uvitec).

#### RT-PCR

Total RNA was extracted from fibroblast cells by using miRNeasy Mini Kit (QIAGEN) according to the manufacturer’s protocol. About 250 ng of total RNA was used to synthetize the first-strand cDNA using iScript cDNA Synthesis Kit (Bio-Rad). The gene expression levels were detected by using EvaGreen qPCR Mastermix (Bio-Rad) and performing real-time PCR on the CFX96 C1000 Touch Real-time PCR system (Bio-Rad) with the following PCR conditions: 98 °C for 30 s, followed by 39 cycles of 98 °C for 2 s and 60 °C for 5 s, and then heating from 70 to 95 °C with either 0.5 °C increments, 5 s/step. Primers for Nav2 (PrimerBank ID 350276221c1 forward: 5'-ACTGGGCCAATCATTACCTAGC-3', reverse: 5'-CGCCATCTGTCACATCTTGCT-3' and PrimerBank ID 350276221c3 forward: 5'-GGTCCTACCGCGAGGGTAT-3', reverse: 5'-TGGCTGCGTCGGTTGTTAG-3') were obtained from the public database PrimerBank (https://pga.mgh.harvard.edu/primerbank/). Differential expression was determined by the 2^−DDCT^ method using GAPDH and ATP5F1 as the internal control.

For flies, RT-PCR was performed as previously described [18] with the following changes. All-In-One 5X RT MasterMix (abm #G592), iTaq Universal SYBR Green Master Mix (BioRad #1,725,120), and a BioRad C1000 Touch Cycler were used. Primers for *sick*: forward: 5'- CACAATTTCCGATGGGTGCTC-3', reverse: 5'- CCTCGGCCCAATGGTTACAT-3'; for *rp49* a housekeeping gene: forward: 5'-TGTCCTTCCAGCTTCAAGATGACCATC-3', reverse: 5'-CTTGGGCTTGCGCATTTGTG-3'.

### Wound Healing Assay

Fibroblasts were seeded in a culture-insert (ibidi culture-insert 2 well, IBIDI) at a density of 2 × 10^4^ cells per well. After allowing the cells to attach and reach confluence, the culture-insert was removed and provided with fresh medium. Migration was documented by taking sequential digital photographs of the gap using an automated microscope (Nikon TiE). Wound area closure was then quantified with ImageJ software, by applying fine edges and sharpen processing tools and analyze particles tool.

### Cell Morphology Analysis

To assess the morphology of cells, fibroblasts were fixed by adding 200 μl of 10% neutral buffered formalin (05‐01005Q, Bio‐Optica) for 5 min at room temperature. After three washings in phosphate‐buffered saline (PBS), cells were permeabilized with Triton X‐100 0.3% in PBS for 5 min, blocked with 1% BSA in PBS for 2 h and then incubated with 0.17 µM Alexa Fluor® 555 Phalloidin (ThermoFisher Scientific) in PBS + 1% BSA for 30 min. Cells were rinsed 3 × with PBS and mounted with Fluoroshield with 4′,6-diamidino-2-phenylindole (DAPI; Sigma-Aldrich) to stain cell nuclei before imaging. Image acquisition was performed using a laser scanning confocal microscope Leica SP8 (Leica Microsystems). Image analysis was performed using Leica and ImageJ software to detect and count cells with filopodia-like cell protrusions. In total, 200–250 cells of the donor and controls were analyzed. Data from control cells were pooled together for statistical analysis.

### Drosophila Immunostaining

Immunostaining of fly larval and adult brains was conducted as described [19]. In short, the dissected samples were fixed in 4% paraformaldehyde (PFA) followed by blocking in 0.2% PBST with 5% normal goat serum. Primary antibodies used: Mouse anti-Repo (DSHB: 8D12) 1:50, Rat anti-Elav (DSHB: 7E8A10) 1:500, Secondary antibodies used: Anti-Rat-647 (Jackson ImmunoResearch, 112–605-003) 1:1000, Anti-Mouse-488 (Jackson ImmunoResearch, 115–545-062) 1:1000. Samples were thoroughly washed with 0.2% PBST and mounted on a glass slide using Fluoromount-G (Southernbiotech, 0100–20). The samples were scanned using a laser confocal microscope (Zeiss LSM 880), and images were processed using ImageJ.

### Drosophila Behavioral Assessment

Twelve-day-old flies were used for behavioral assessment. Climbing (negative geotaxis) assays were performed essentially as previously described [20]. Flies were transferred to a clean, empty vial and given 5–15 min to habituate before being tapped to the bottom of the vial and assessed for a negative geotaxis response. Climbing distances were measured at 15 cm in a given time (20 s as maximum). All flies were reared at 25 °C. Flies were transferred into a fresh vial every 3 days.

Heat-induced seizure assays were performed as previously described [21]. Flies were transferred to a clean vial and allowed to habituate for 5–15 min before the vial was immersed in a 42 °C water bath for 30 s. Seizures were defined as failure to maintain an upright posture combined with wing fluttering, leg twitching, and sometimes abdominal curling. The percentage of seizing flies at 30 s was calculated. After immersion for 30 s, the vial was taken out of the water to allow recovery. The recovery time of individual flies to an upright posture was measured.

### Drosophila stocks

The following stocks were used in this study:

**Table:** 

Fly line	Genotype	Source
*sick*^*T2A−GAL4*^	*y*^*1*^ w^*^; Mi{Trojan-GAL4.0}sickMI08398-TG4.0/SM6a	BDSC #76,195
*sick-Df*	*w[1118]; Df(2L)ED1303, P{w[*+ *mW.Scer\FRT.hs3]* = *3'.RS5* + *3.3'}ED1303/SM6a*	BDSC #8679
*UAS-mCherry.NLS*	w[*];; P{w[+ *mC]* = *UAS-mCherry.NLS}3*	BDSC #38,424
*UAS-mCD8-RFP*	w[*]; P{y[+ *t7.7] w[*+ *mC]* = *10XUAS-IVS-mCD8::RFP}attP40;*	BDSC #32,219
*Canton S*	Wild type	Bellen Lab

### Data Presentation and Analysis

Statistical analysis was performed using GraphPad software (GraphPad Prism v9.0; GraphPad Software, USA). Data were presented as representative images or as mean ± SEM. A statistical analysis of data was performed with Student’s *t*-test and/or ANOVA.

## Results

### Identification of NAV2 Variants and Bioinformatic Analyses

**Fig. 1:**
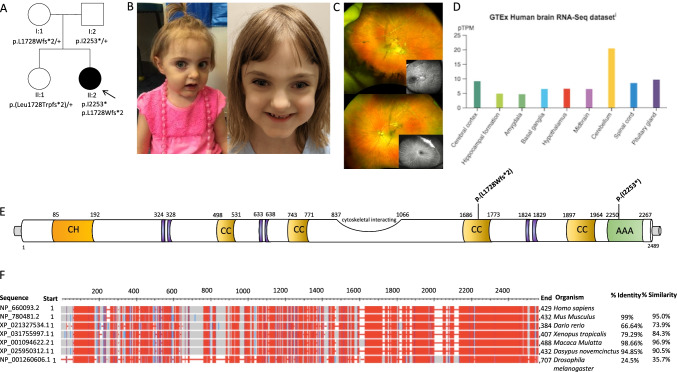
Clinical and genetic findings of the *NAV2*-related neurodevelopmental disorder in the family of the proband. (A) Pedigree of the family showing the affected individual (shaded). + represents the reference allele. (B) Craniofacial dysmorphism of the affected subject (at the age of 3 years left panel and 7 years right panel) including deep set eyes, upslanting palpebral fissures, bulbous nasal tip, thin upper lip, and dimple and broad chin. (C) Retinal fundus photograph of the right and left eyes illustrating aberrant retinal vasculature with a subclinical retinal detachment in the right eye and retinal neovascularization left eye. Ultra-widefield fluorescein angiography of the right and left eyes demonstrating retinal ischemia and retinal neovascularization (inset). (D) RNA-seq tissue data generated by the Genotype-Tissue Expression (GTEx) project and reported as mean pTPM (protein coding transcripts per million), corresponding to the values of the different individual samples for respective subregion. Cerebellum has the highest expression (pTPM 20.4). (E) Depictions of the pathogenic variants p.(L1728Wfs*2) and p.(Ile2253*) and protein domains (CH, calponin homology domain; cytoskeletal interacting domain or CSID; CC, coiled coil domain; AAA, AAA-ATPase domain; purple indicates poly-Proline, Serine, and Lysine regions). (F) NAV2 protein sequences of different species based on the constraint-based alignment tool COBALT. The red colour indicates highly conserved protein regions among species and blue indicates less conserved ones. Percent of identity (indicating the percentage of the orthologous sequence matching the Human sequence according to the Ensembl database) and the percent of similarlity have been calculated using the EMBOSS Needle tool

Stepwise filtering of ES analysis retained two compound heterozygous variants in *NAV2* (NM_001244963.2): c.5179_5180delAG, p.(Leu1728Trpfs*2) and c.6757delA,p.(Ile2253*) in a female with developmental delay and a diagnosis of cerebellar hypoplasia and dysplasia. Sanger sequencing confirmed co-segregation of the variants with the disease within the family. The unaffected parents and a healthy sibling all carry one of the two variants (Fig. 1A). Both variants were absent from the gnomAD database (https://gnomad.broadinstitute.org) and classified as of unknown significance according to the ACMG criteria. Remarkably, *NAV2* is a *loss-of-function* intolerant gene (pLI = 1) as no homozygous *loss-of-function* variants are reported in the gnomAD database (last accessed 05 May 2021; Supplemental Table 1) [22]. Furthermore, *NAV2* is predicted to be potentially associated with a recessive condition according to a linear discriminant analysis (LDA) score of − 0.003 (< 0.5 corresponds to a “likely recessive” class) by the DOMINO algorithm [23]. Moreover, NAV2 is abundantly expressed in the human CNS, especially in the cerebellum (Fig. 1D), and it is conserved across different species (Fig. 1F), suggesting that *loss-of-function* in humans may have detrimental effects similar to the *Drosophila*, *Caenorhabditis elegans*, and mouse.

**Table 1:** Phenotypic comparison between human and mouse phenotype due to NAV2 deficiency

Trait	Human	Mouse
Age at onset	Congenital	Congenital
Clinical findings	Broad based gait	Ataxic gait*
	Developmental delay	ND
	Oculomotor apraxia	ND
	Craniofacial dysmorphism	ND
Brain findings		
Cerebellum	Vermis hypoplasia, cerebellar cortical dysplasia	Hypoplasia, vermal foliation defects*
Corpus callosum	Hypodysgenesis	Hypodysgenesis
Anterior commissure	Agenesis	Normal
Pons	Hypoplasia	Normal
Medulla	Normal	Normal
Thalamus/Hypothalamus	Normal	Hypoplasia
Olfactory system	Olfactory bulb agenesis	Impaired olfactory acuity**
Eye anomalies	Optic nerve hypoplasia	Optic nerve hypoplasia**
Congenital heart defects	Dysplastic aortic, pulmonary, mitral, and tricuspid valves	ND

^*^Findings from McNeill et al. (2011) in the *Nav2* (*unc-53H2*) hypomorphic mutant mouse

^**^Findings from Peeters et al. (2004) in the *Nav2* (*unc-53H2*) hypomorphic mutant mouse

*ND*, not determined

Interrogation of GeneMatcher and additional Matchmaker platforms for *NAV2* failed to identify additional cases with biallelic *loss-of-function* variants and/or similar phenotypes. Finally, no biallelic *loss-of-function* variants in this gene were found in the genomic datasets of the 100 K Genome Project.

ES from the affected individual did not reveal pathogenic or likely pathogenic variants in any known disease gene or other gene. The biallelic *loss-of-function* variants in *NAV2* therefore emerged as the most likely cause for the disease, given the severity of the biallelic variants (predicted to undergo nonsense mediated decay), and the known role of Neuron Navigator 2 across different species.

### Clinical and Neuroradiological Features Associated with Biallelic NAV2 Variants

The affected individual was the second child to healthy and nonconsanguineous parents of Caucasian ancestry (Fig. 1A, II.2). Family history was unremarkable. Mother’s pregnancy was complicated by oligohydramnios and gestational diabetes treated with diet. Antenatal history was remarkable for frequent atrial ectopy, polyvalvular heart disease with atrioventricular valve regurgitation, and pericardial effusion in the fetus. She was treated with digoxin for suspected supraventricular tachycardia. She was born by cesarean section at 38 weeks gestation. Growth parameters at birth indicated weight 2915 g (− 0.96SDs), length 47.6 cm (− 1.21SDs), and occipital frontal circumference (OFC) 33 cm (− 1.19SDs). Postnatal echocardiogram showed a common atrium, an unicommisural aortic valve with mild stenosis, bicuspid pulmonary valve with mild stenosis, and mildly thickened tricuspid and mitral valves without stenosis. Head ultrasound showed thin corpus callosum with suspected vermian dysplasia. Brain MRI at birth showed marked hypoplasia of the corpus callosum with small cerebellar vermis and dysmorphic appearance of the pons. Renal ultrasound and EEG were unremarkable.

**Fig. 2:**
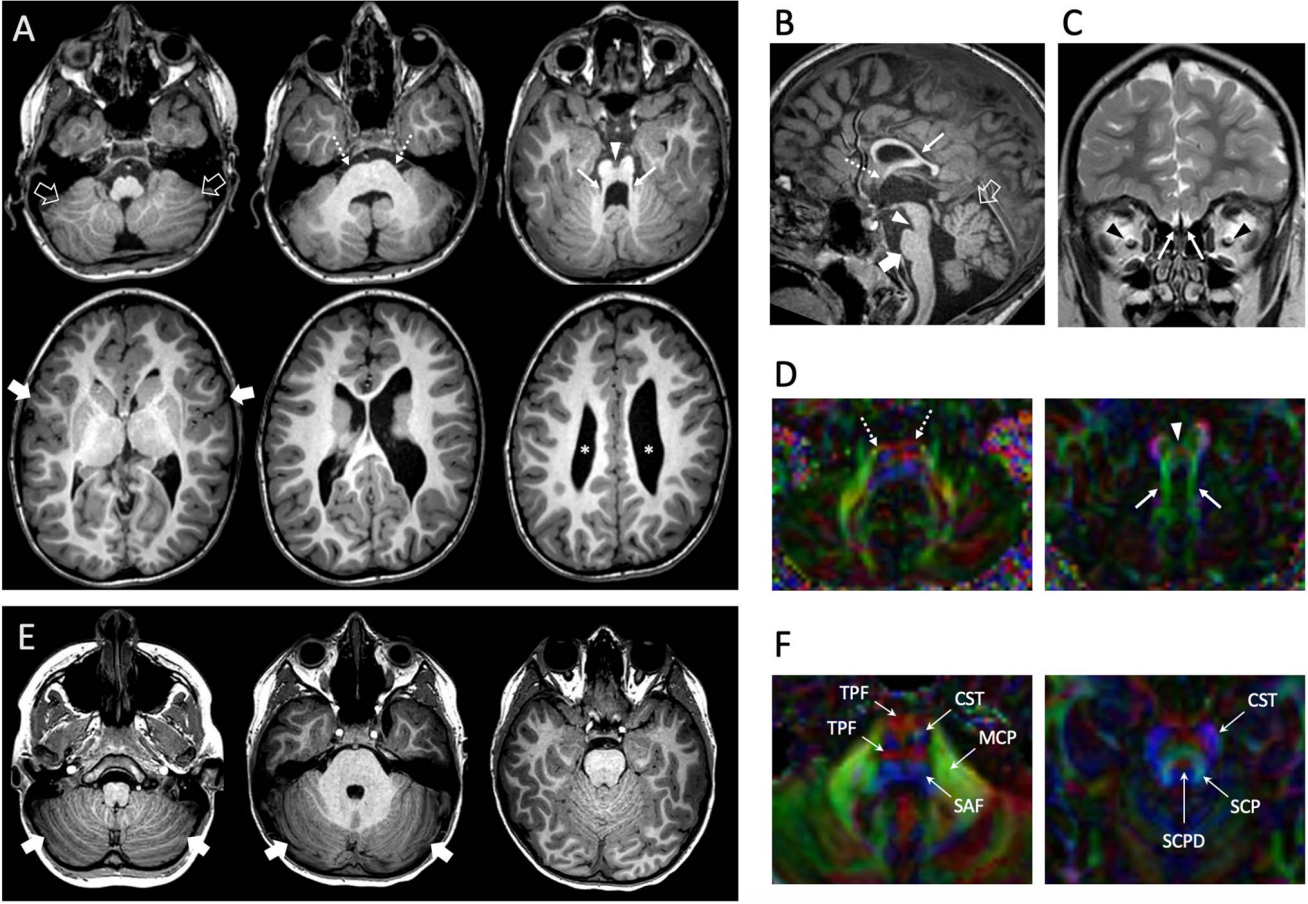
Neuroimaging features of the NAV2-related neurodevelopmental disorder in the proband. Brain MRI of the patient performed at 6 years of age (A–D) and of an age-matched control subject (E–F). A) Axial reformatted 3D T1-weighted images reveal abnormal cerebellar foliation fissuration, more pronounced at the level of the inferior cerebellar hemispheres (empty arrows). The pons is flattened and slightly asymmetric (dotted arrows). The superior cerebellar peduncles are thinned and splayed (arrows) with associated narrowing of the isthmic region (arrowhead) leading to a molar tooth-like appearance of the midbrain. Note the diffuse cortical dysgyria, with prevalent insular involvement (thick arrows), and the asymmetric enlargement of the lateral ventricles (asterisks). (B) Sagittal reformatted 3D T1-weighted image shows the hypoplasia and dysplasia with mild upward rotation of the cerebellar vermis, and prevalent involvement of the anterior and superior posterior lobes (empty arrows). There are also corpus callosum hypodysgenesis (arrow), agenesis of the anterior commissure (dotted arrow), narrow isthmus (arrowhead), and small pons (thick arrow). Note the small optic nerve chiasm. (C) Coronal T2-weighted image demonstrates the agenesis of the olfactory bulbs (arrows) and mild hypoplasia of the optic nerves (arrowheads). (D) Diffusion tensor imaging, axial color-coded fractional anisotropy (FA) maps reveal small asymmetric corticospinal tracts at the level of the pons (dotted arrows) and horizontal course of the superior cerebellar peduncles (arrows) with preservation of their decussation at the midbrain level (arrowhead). (E) Axial reformatted 3D T1-weighted images show the normal size and morphology of the cerebellar hemispheres. The cerebellar folia run parallel to the calvarium (onion-like configuration; thick arrows). (F) Axial color-coded FA maps, magnified view at 2 brainstem levels (middle pons and middle midbrain). Conventional color scheme: blue (inferior-superior), green (anteroposterior), and red (left–right). CST, corticospinal tract; MCP, middle cerebellar peduncles; SCP, superior cerebellar peduncles; SCPD, superior cerebellar peduncles’ decussation; SAF, somatosensory ascending fibers; TPF, transverse pontocerebellar fibers

**Fig. 3:**
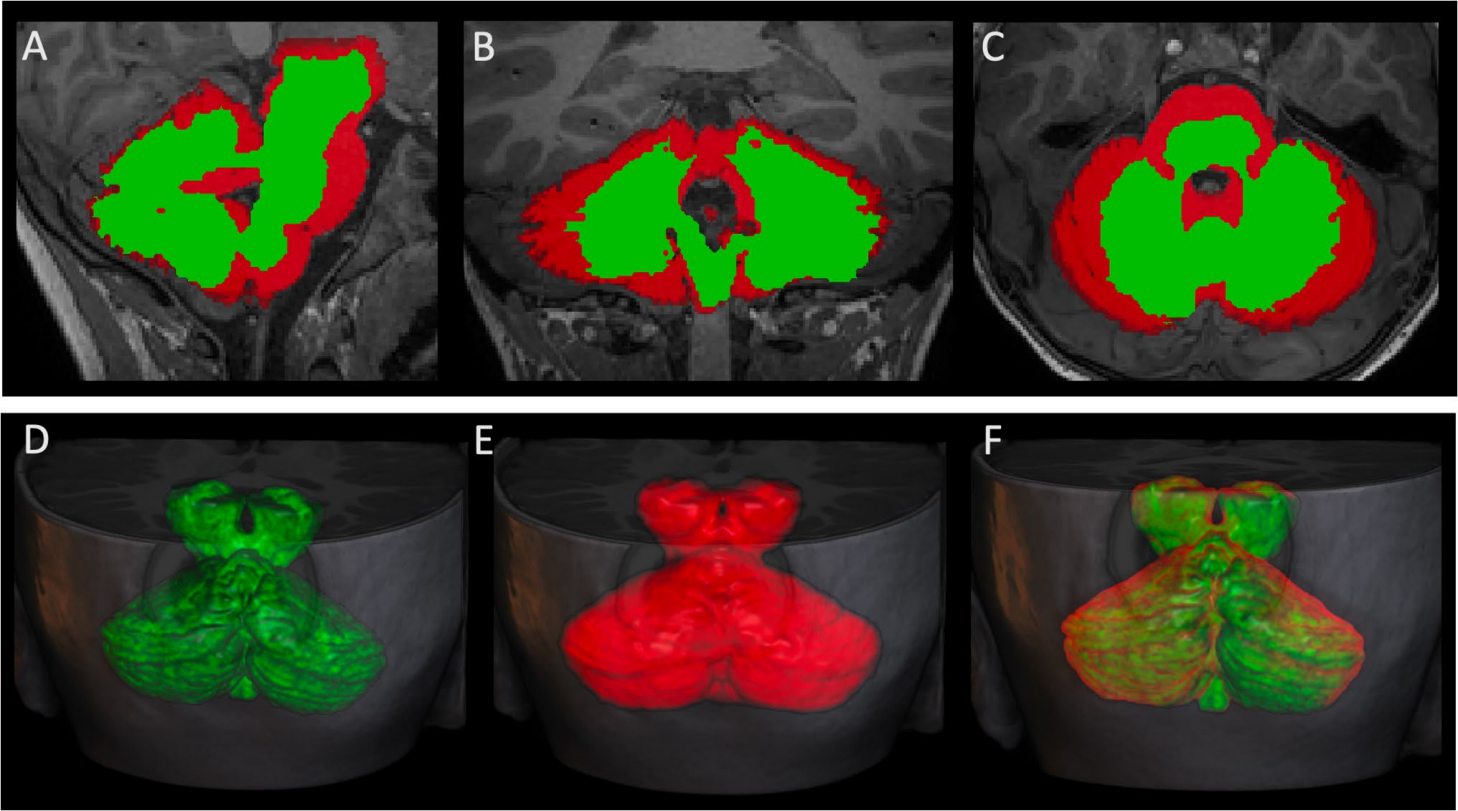
Comparison of the cerebellar volumes of the patient with an age-matched healthy subject. (A–C) Segmentation of the cerebellar volumes of the patient (green maps) and of an aged-matched control subject (red maps) overlayed on sagittal (A), coronal (B), and axial-reformatted (C) 3DT1-weighted images. (D–F) Volumetric reconstructions of cerebellar segmentations of the patient (green cerebellum, D), of the control subject (red cerebellum, E), and of the fusion of both cerebellar volumes (F) overlayed on 3D T1-weighted images. Note that the volume of both the cerebellar hemispheres and vermis of the patient is smaller compared to the age-matched control

At 4 weeks of life, she presented with cardiogenic shock requiring cardioversion and intubation. She had severe global ventricular dysfunction and atrial flutter. At 5 weeks of age, she underwent mitral and tricuspid valve repair, open aortic valvotomy, and pericardial patch closure of secundum atrial septal defect. Her initial ophthalmological evaluation indicated possible motor apraxia and mild optic atrophy. Brain MRI repeated at 3 and 6 years of age revealed marked cerebellar vermis hypoplasia, bilateral cerebellar foliation defects, pontine hypo-dysplasia, splayed thin superior cerebellar peduncles with a molar tooth-like configuration, corpus callosum hypodysgenesis, absent anterior commissure, diffuse dysgyria, agenesis of the olfactory bulbs, mild optic nerve hypoplasia, and enlarged dysmorphic lateral ventricles (Fig. 2). Cerebellar morphometry data of the patient were compared with normal values derived from an in-house database of controls [24]. The transverse cerebellar diameter (89 mm), the cranio-caudal diameter of the vermis (27.5 mm), and the antero-posterior diameter of the vermis (20 mm) were below 2SD compared with age-matched controls. The results of volumetric comparison of the cerebellum of the patient with an age-matched healthy subject performed with the SUIT toolbox of SPM 12 [25] are displayed in Fig. 3. She had significant motor delays; however, her cognitive functioning was assessed to be normal when assessed using Wechsler Preschool and Primary Scale of Intelligence (WPPSI-IV) at 3 years of age. She struggled with motor delays and, despite some improvements with physiotherapy and occupational therapy, continued to need assistance with most activities of daily living. Her formal developmental assessment at the age of 6 years and 8 months revealed gross and fine motor delay and both receptive and expressive language impairment. She subsequently developed decreased vision in both eyes and was found to have mild optic atrophy, atypical chorioretinal scarring, retinal ischemia, retinal neovascularization, and retinal detachment (Fig. 1C). She subsequently had panretinal photocoagulation laser of both eyes. Her physical examination at 7 years of age showed microcephaly with OFC of 48 cm (− 2.8 SDs) with normal weight and height. Mild dysmorphic features were seen including deep-set eyes, upslanting palpebral fissures, bulbous nasal tip, thin upper lip vermilion, and dimpled and broad chin (Fig. 1B). She also had hypotonia and impairment of voluntary, saccadic eye movements (Supplemental material Videos 1, 2).

### Histopathological Features of Nav2 Mutant Mice and Comparison with Human Phenotype

**Fig. 4:**
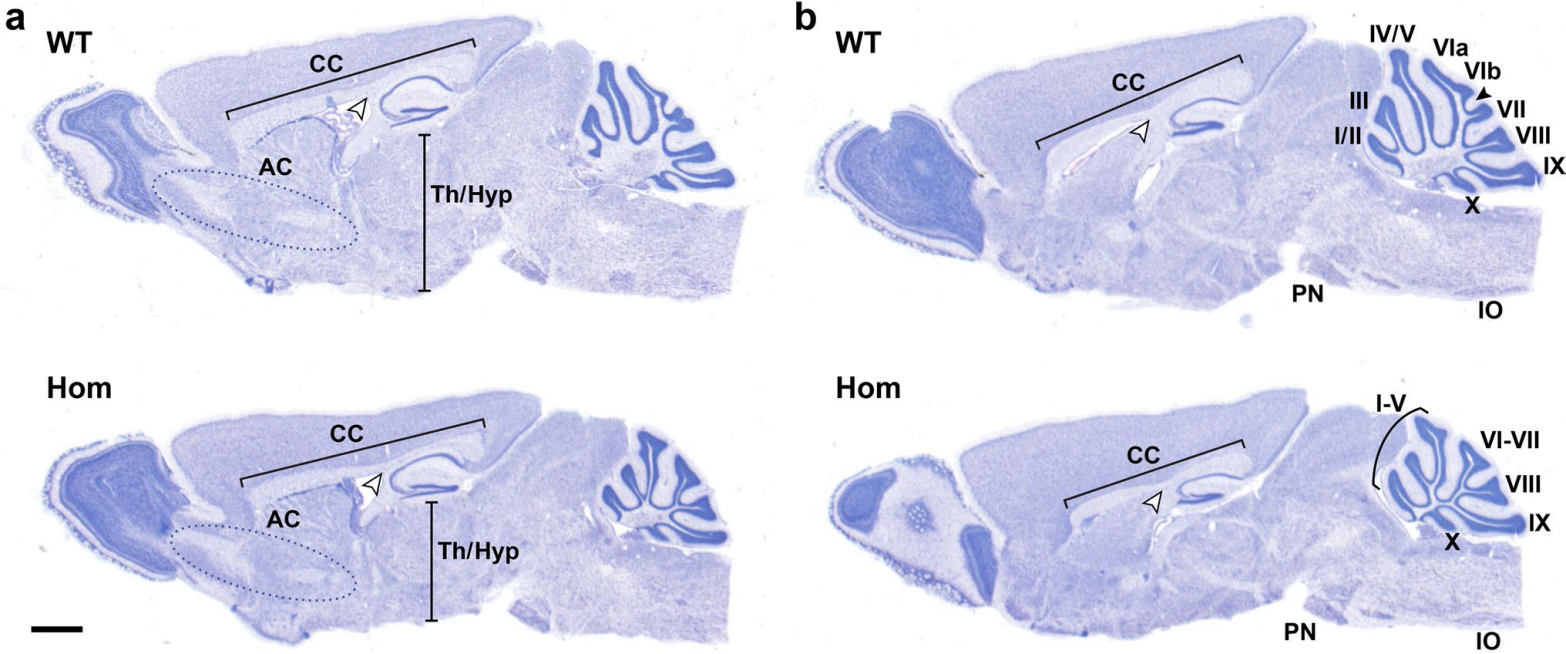
Brain and cerebellar abnormalities in the Nav2 hypomorphic mutant mice. Medial sagittal sections (panels **a** and **b**; panel **b** closest to midline) from a wild-type (WT) and homozygous hypomorph (HOM) show an overall reduction in cerebellar size, and a reduction in overall development of VIa,VIb/VII with absence of the intercrural fissue (arrowhead) in the HOM. In other brain regions, abnormalities noted in the HOM include thinning of the corpus callosum and a reduction in the size of the regions encompassing the thalamus/hypothalamus (Th/Hyp). The pons and medulla appear normal in size in both genotypes, with the pontine nucleus (PN) and inferior olive (IO) shown for reference. The cortex and tectum, anterior commissure (AC), and olfactory bulb showed no obvious dysmorphogenesis. Scale bar: 1 mm

Analysis of the mouse model confirmed an overall reduction in cerebellar size, abnormal foliation in the I-V region along with impaired development of VIa and VIb/VII lobes (Fig. 4, lower panels, Supplemental Fig. 1D). Previously unreported abnormalities in other brain regions included thinning of the corpus callosum and a reduction in the size of the thalamus and hypothalamus. There were no major abnormalities of the cerebral cortex, brainstem, anterior commissure, and olfactory bulbs (Fig. 3). A detailed comparison between the human and mouse phenotype is summarized in Table 1 and depicted in Supplemental Fig. 1.

### NAV2 Deficiency Perturbs Cell Migration and Cytoskeleton Organization

**Fig. 5:**
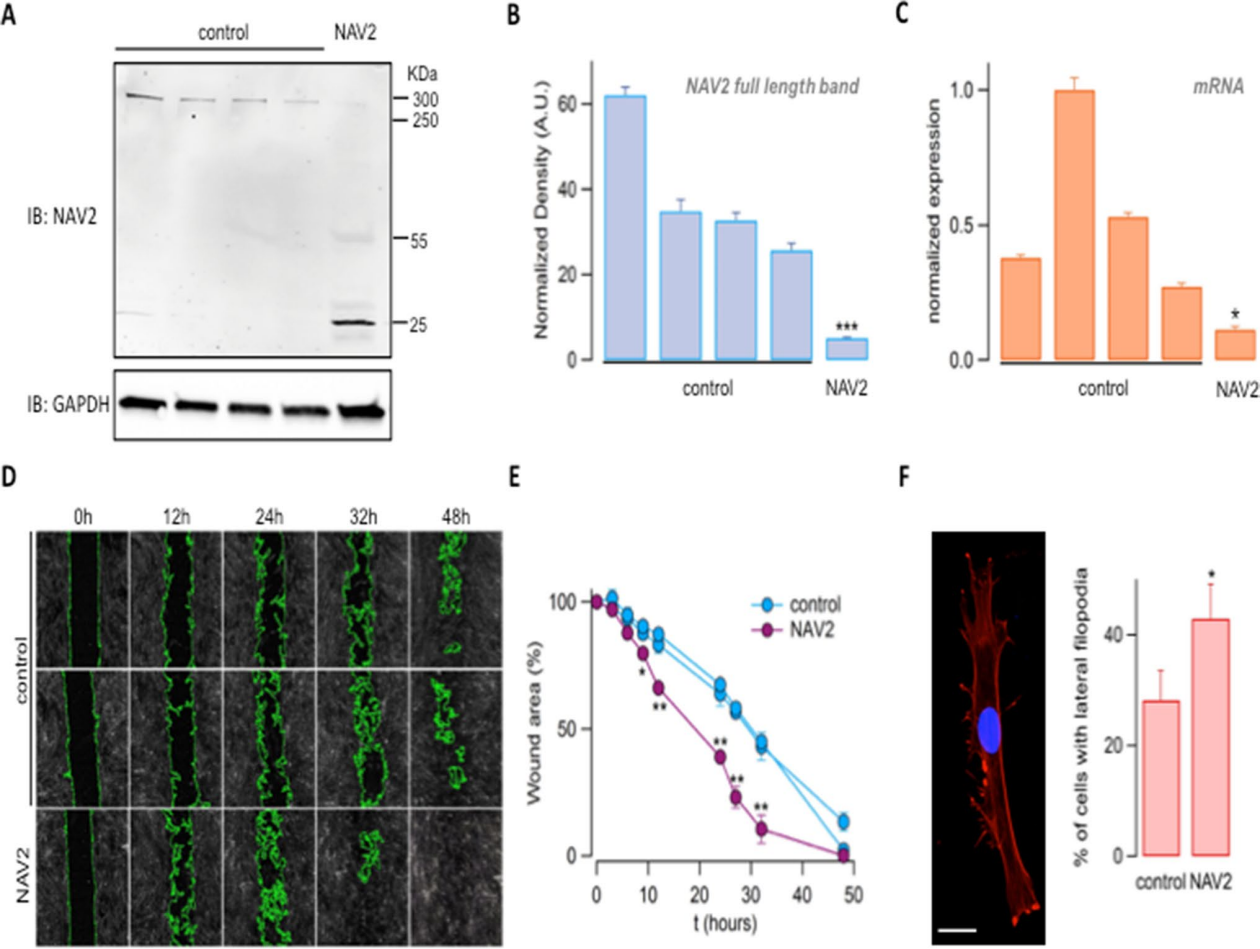
Cellular phenotype associated with the genetic loss of Neuron Navigator 2 in the NAV2 compound mutant patient. (A–C) Analysis of NAV2 protein and mRNA expression in fibroblasts obtained from 4 healthy individuals and the “NAV2 patient.” (A) Representative Western blot experiment showing immunodetection of NAV2 (top) and GAPDH (bottom) in the indicated fibroblasts lysates. (B) Bar graph showing the densitometric analysis of the upper band, corresponding to the full-length protein (2830 KDa); data are normalized for GAPDH expression. *N* = 3, *** p < 0.001 (ANOVA with Tukey’s post hoc test). (C) Bar graph showing NAV2 mRNA quantification by real time PCR. *N* = 3, ** *p* < 0.01 (ANOVA with Tukey’s post hoc test). (D and E) Analysis of cell migration by wound healing assay. Representative images (D) and analysis (E) of wounded areas of confluent fibroblasts at different time points. Wound edges, detected by image segmentation analysis, are outlined in green. *N* = 3; * *p* < 0.05, ** *p* < 0.01. (F) Representative image and summary graph showing results from cell morphology analysis. Fibroblasts were fixed, stained with phalloidin (red) and Hoechst 33,342 (blue) and analyzed by confocal microscopy. Scale bar = 10 µm. 200–250 cells/donor were analyzed. * *p* < 0.05

We first assessed the expression of the mutant NAV2 by western blot analysis on patient and control fibroblasts. As expected, we found an intense band corresponding to a molecular weight of ~ 283 kDa in total lysates from four different control fibroblasts that was almost totally absent in lysates from patient fibroblasts (Fig. 5A, B). Some faint and one intense bands of lower molecular weights (< 55 kDa) appeared in patient lysates, most likely representing protein fragments resulting from degradation of the truncated NAV2 protein (Fig. 5A). This finding was consistent with the mRNA quantification by real-time RT-PCR that showed a significant reduction compared to controls, indicating that mutant NAV2 mRNA undergoes nonsense-mediated decay (Fig. 5C).

We next sought to assess the effect of this severe NAV2 deficiency on cell migration. In the wound healing assay, patient fibroblasts showed perturbed migration compared to controls, as demonstrated by the increased quantification of the wound area closure at different time points (Fig. 5D, E). In addition, we noticed that patient-derived fibroblasts also displayed a peculiar morphology compared to controls, characterized by the presence of several cell protrusions. Indeed, phalloidin staining and confocal microscopy imaging and analysis revealed an increased proportion of lateral filopodia-rich cells (Fig. 5F), a phenotype likely caused by abnormal cytoskeleton dynamics due to NAV2 genetic deficiency.

### The Drosophila NAV2 ortholog, sick, is enriched in the developing and adult CNS and mutants are semi-lethal

**Fig. 6:**
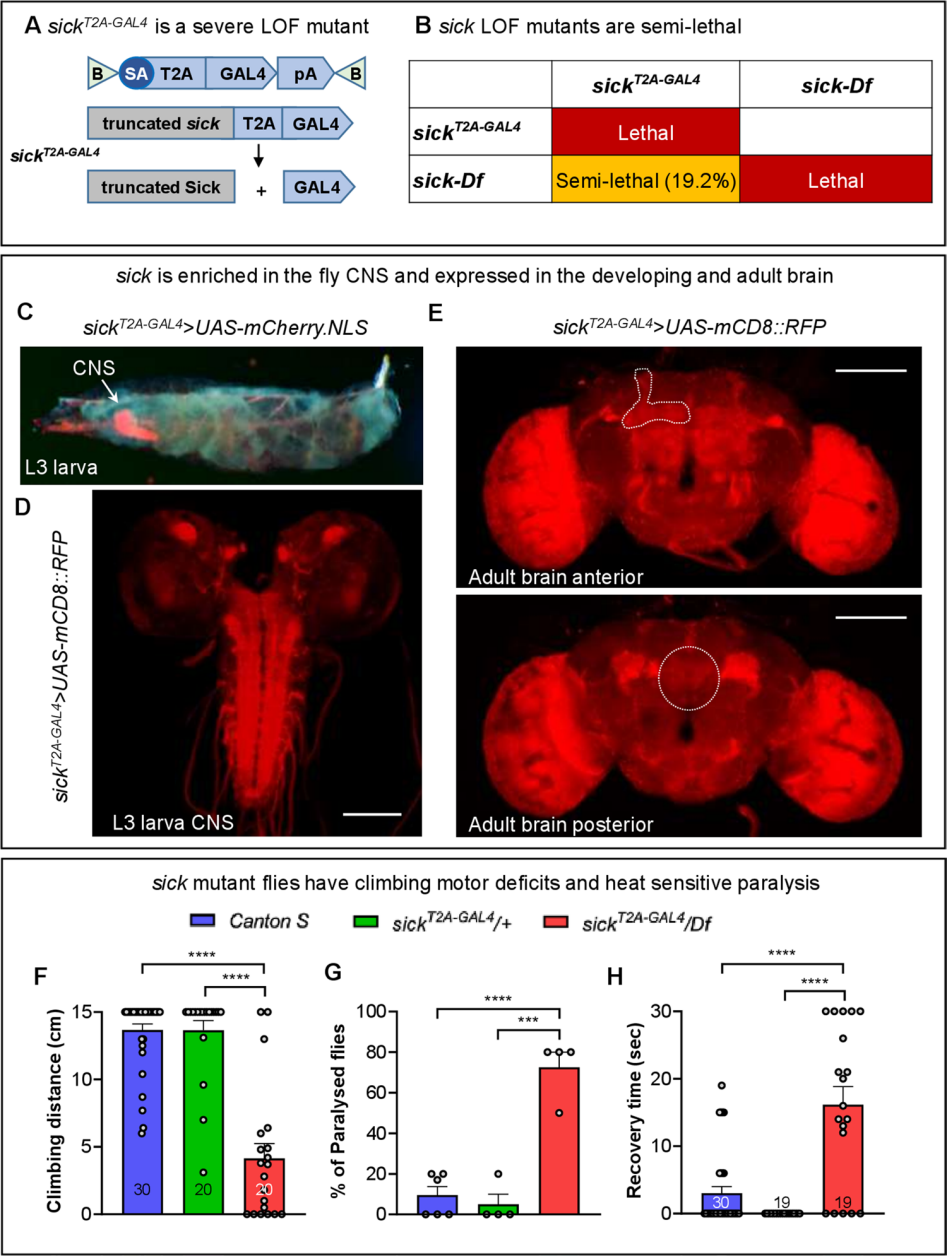
The NAV2 ortholog in *Drosophila*, sick, is expressed in brain and mutants are semi-lethal with motor defects and heat-sensitive seizure-like behavior. (A) Schematics of *sick*^*T2A−GAL4*^ acting as a gene trap: the insertion of SA-T2A-GAL4-polyA cassette leads to generation of truncated Sick and expression of GAL4 under the control of the regulatory sequences of the *sick*. (B) Complementation tests of *sick*^*T2A−GAL4*^ with a corresponding deficiency (*Df(2L)ED1303*). (C–E) Gene expression of *sick* based on *sick*^*T2A−GAL4*^; *UAS-mCD8::RFP* flies. Whole third instar larva (C), third instar larval brain (D), and adult brain (E) are shown. Note expression in the mushroom bodies (upper panel, dashed lines), olfactory glomeruli (upper panel, dashed ellipse), and antennal mechanosensory and motor center (upper panel, dashed ellipse). Scale bars = 100 µm. (F) Climbing assessment of *sick*^*T2A−GAL4*^*/Df* flies in a negative geotaxis assay reveals motor deficits in *sick* mutants that are 12 days old. *N* are shown within the bars. Unpaired *t* tests, **** *p* < 0.0001. (G) *sick* mutants display heat-induced seizures in a 42 °C water bath (30 s). (H) Time to recover. *N* are shown within the bars. Unpaired *t* tests, *** *p* < 0.001, **** *p* < 0.0001

To determine the role of NAV in development and neuronal function, we explored the phenotypes associated with loss of the NAV orthologue in flies. The fruit fly has a single Neuron Navigator gene called *sick*, orthologous to all three NAV(1/2/3) family members in humans. Based on multiple orthology prediction^26^, *sick* is most orthologous to NAV2 (DIOPT score is 9/16, Supplemental Fig. 2A). Moreover, both the CH and AAA domains are highly conserved in both Sick and NAV2 (Supplemental Fig. 2B). We previously generated *sick* mutants as part of the Gene Disruption Project^27^. This allele, *sick*^*T2A−GAL4*^, was generated by introduction of an artificial exon containing a splice acceptor-T2A-GAL4-polyA cassette between exons 10 and 11 and is predicted to act as strong loss-of-function mutation because of the presence of a poly-A tail (Fig. 6A, Supplemental Fig. 2C). The *sick*^*T2A−GAL4*^ allele also produces a GAL4 in the same spatial and temporal pattern as the *sick* gene reflecting the endogenous expression. The *sick*^*T2A−GAL4*^ mutants are homozygous lethal and but are semi-lethal (only 19.2% of flies eclose) when *in trans* to deficiency, *Df(2L)ED1303* (Fig. 6B), suggesting that the *sick*^*T2A−GAL4*^ chromosome may carry a modifier. In the *sick*^*T2A−GAL4*^*/ Df* flies, the transcript levels are decreased to 15.2% of wild-type controls (*Canton S*) (Supplemental Fig. 2 D).

In order to determine the expression pattern of *sick*, we generated *sick*^*T2A−GAL4*^; *UAS-mCherry.NLS* flies. The GAL4 drives the expression of nuclear mCherry. Third instar larvae exhibit robust expression of the reporter in the CNS with sparse expression in the trachea (Fig. 6C). To reveal the projections of the neurons that express *sick*, we crossed the *sick*^*T2A−GAL4*^ mutants to a membrane bound reporter (*UAS-mCD8::RFP*)*.* In the third instar larvae, *sick* is enriched in motor neurons of the ventral nerve cord as well as the mushroom body neurons (Fig. 6D). In the adult brain, *sick* is widely expressed, but it shows notable enrichment in the optic lobes, mushroom body, and antennal mechanosensory and motor center of the fly brain (Fig. 6E). To determine if *sick* is restricted to neurons or is also present in the glia, we crossed the *sick*^*T2A−GAL4*^ mutants to *UAS-mCherry.NLS* and examined third instar larva brain and adult brains that are co-stained with nuclear markers for neurons (Elav) and glia (Repo). As shown in Supplemental Fig. 3, *sick* is expressed in both neurons and glia in the developing CNS (Supplemental Fig. 3A) and adult brain (Supplemental Fig.3 B).

### Surviving Sick Mutants Show Climbing and Heat-Induced Seizures

A previous study documented that *sick* mutants exhibit axonal growth defects during development [13], but nothing was reported in adult flies. To determine the functional consequences of loss of *sick* in adult flies, we conducted neurobehavioral assessments. We found that the climbing ability of 12-day-old *sick*^*T2A−GAL4*^*/Df* flies is severely impaired compared to control flies (Fig. 6F). These data indicate that the motor function of *sick* mutant flies is impaired.

Hyperthermia increases the intrinsic excitability of both excitatory and inhibitory neurons, and the dysregulated synaptic activity can induce febrile seizures [21, 26, 27]. To assess if loss of *sick* also leads to altered synaptic activity, we performed heat induced seizures by subjecting flies to 42 °C for 30 s [21]. We observed that *sick*^*T2A−GAL4*^*/Df* flies exhibit a rapid onset of seizure like behavior (Supplemental material Videos 3, 4). Over 75% of *sick*^*T2A−GAL4*^*/Df* flies exhibit heat induced seizures and they require about 15 s to recover once returned to room temperature (Fig. 6G, H). Hence, the data indicate dysregulated synaptic activity in *sick* mutants.

## Discussion

In this study, we report biallelic truncating variants in *NAV2* associated with a novel human neurodevelopmental phenotype characterized by vermis hypoplasia and cerebellar cortical dysplasia as well as other brain malformation. The *NAV2* gene encodes multiple transcripts and proteins based on alternate promoter usage and splicing. The NAV2 transcript variant 5 encodes the largest isoform of 2488 amino acids. Full-length NAV2 proteins contain several putative functional domains, including a calponin-homology (CH) domain at the N-terminus, several coiled-coil regions, a cytoskeletal interacting domain (or CSID), and an ATP/GTP nucleotide-binding site (AAA-domain) at the C-terminus (Fig. 1E). We functionally investigated the impact of compound heterozygous variants identified in this study [NM_001244963.2: p.(L1728Wfs*2) and p.(Ile2253*)] on a cellular level, and showed that the variants cause an almost total absence of mRNA production and protein expression in patient-derived fibroblasts, suggesting that they cause a severe loss of function.

The brain and cerebellar phenotype reported in this study is partially overlapping to the one observed in the *Nav2* mouse model. Indeed, hypomorphic mutant mice lacking the full-length *Nav2* transcript exhibit ataxia with reduced volume and abnormal foliation of the vermis mostly affecting folia VI-VII [14]. *Nav2* hypomorphic mutants show the same cerebellar malformations after 20 backcrosses as described previously by McNeill et al. (2011). In both the earlier and present study, hypoplasia of folia VI-VII and loss of the intercrural fissure were found in all *Nav*2 mutants and none of the wild-type controls (Fig. 3, Supplementary Fig. 1 and McNeill et al., 2011). In the earlier study, an effect on anterior folia was observed in 67% of *Nav2* mutants but was not observed in wild-type mice. The malformations seen in the *Nav2* mutant are distinct from spontaneous malformations in the cerebellar vermis of wild-type C57BL/6 mice which largely affect folia VIII-IX [28, 29]. Similar to the *Nav2* mutant mouse, our patient displays hypoplasia and dysplasia of the vermis with prevalent involvement of the anterior and superior posterior lobes. In *Nav2* mutant mice, there is a delay in the disappearance of the external germinal layer resulting from impaired granule cell migration toward the interior of the cerebellum during development. The inability of granule cells to extend neurites/parallel axon fibers and migrate properly was observed in cultured explants and dissociated granule cell cultures from mutants [14]. This finding could explain the abnormal foliation observed in the patient, suggesting that abnormal granule cell migration and axonal outgrowth defects lead to cerebellar cortical dysplasia.

Overall, the cerebellar features due to NAV2 genetic deficiency are further corroborated by our comparison between the histopathological analysis of the mutant mice and the neuroimaging features of our patient, underscoring an impairment of cerebellar development predominantly affecting the vermis and the correct folia orientation. Moreover, the presence of oculomotor apraxia in the patient suggests disruption of cerebro-cerebellar circuits that are crucial for the control of voluntary eye movements. This is not surprising given the broad expression of NAV2 across the entire CNS [8] and its role in the development of other brain structures such as cranial nerves [30]. A novel finding emerged from the re-evaluation of the mouse brain histopathological features including corpus callosum hypodysgenesis similar to the patient; this suggests migration and axon elongation defects of midline neuronal circuits/networks that are affected by NAV2. Interestingly, the patient also displayed mild optic nerve hypoplasia and agenesis of the olfactory bulbs and both optic nerve hypoplasia and impaired integrity of olfactory sensory systems have also been observed in the hypomorphic *Nav2* mutant mouse [31]. Moreover, it is known that retinal ganglion cell axons guide the formation of an astrocytic network that in turn dictate the pattern of developing retinal vasculature [32, 33]. Therefore, the aberrant retinal vasculature found in the patient may also be related to inappropriate ganglion cell migration during retinal development leading to subsequent retinal neovascularization and detachment later in life.

The wound healing assay in patient fibroblast cultures revealed a cellular migration defect. This is consistent with the increased staining of filopodia observed with confocal microscopy suggesting an alteration of cytoskeletal architecture that affects migration process. Filopodia are thin membrane protrusions that sense the extracellular environment at their tips using cell surface receptors and promote retrograde flow of actin upon binding with external targets, eventually leading to different cell migration processes such as wound healing and neurite outgrowth [34]. However, substantial differences in terms of migration dynamics and regulatory networks occur between fibroblasts and cerebellar granule cells [35].

It is worth mentioning that impaired proliferation may account for wound healing defect [36]. Since we did not specifically look at proliferation in our wound healing assay, we cannot rule out a possible contribution of abnormal proliferation for the reduced size of the cerebellum as previously suggested [14].

NAV2 may act by facilitating interactions between microtubules and other proteins such as neurofilaments that are key players in the formation and stability of growing neurites, and the aberrant cytoskeleton architecture derived from the loss of NAV2 might lead to secondary microtubule dysfunction [9]. This hypothesis is supported by several imaging features observed in the context of NAV2 deficiency that can be also found in individuals affected with rare tubulinopathies, such as cerebellar dysplasia with foliation defects, brainstem abnormalities, diffuse cortical dysgyria, olfactory bulb agenesis, and asymmetric lateral ventricles [37]. Importantly, only the absence of the characteristic basal ganglia anomalies differentiates these two conditions. This feature might be explained by the different expression profile of the *NAV2* in the CNS. Indeed, brain regions with the most abundant expression included the developing cortex, hippocampus, thalamus, olfactory bulb, and granule cells of the cerebellum. In contrast, expression of *NAV2* in basal ganglia and white matter expression was largely undetectable [8]. Noteworthy, cerebellar cortical dysplasia is also found in a few other NDDs that are frequently autosomal recessive, such as Joubert syndrome and related disorders (JSRDs), Poretti–Boltshauser syndrome, muscular dystrophy (dystroglycanopathies), and Chudley-McCullough syndrome [4, 5]. In these conditions, the association with other medical issues and additional brainstem and/or cerebral imaging features may help the differential diagnosis, as presence of hearing loss, callosal agenesis, fronto-mesial polymicrogyria, and intracranial cysts in Chudley–McCullough syndrome or the molar tooth sign and progressive retinal, kidney, and liver disease in JSRDs.

Studies of the *NAV2* ortholog, *sick*, in *Drosophila* show that it is present in both the mushroom body and the antennal lobes as well as some other neurons. The mushroom body is widely known as the learning and memory center of the flies[38], whereas the antennal lobes integrate olfactory information [39]. A previous study showed that Sick is required for axonal growth of mushroom body neurons and that its loss caused defective mushroom body and ellipsoid bodies [13]. It is worth noticing that the core architecture of mushroom body circuit is strikingly similar to that of the vertebrate cerebellum [40, 41]. Moreover, the *sick* mutants display motor deficits indicating Sick function is important for proper motor output in flies. Taken together, these findings further underscore the conserved function of *NAV2*/*Sick* in brain and cerebellar development throughout several species.

While the affected subject did not display seizure, the sick mutant developed heat-induced seizure. Since the homeostatic plasticity of the brain requires proper wiring of circuits containing excitatory and inhibitory neurons, dysregulated synaptic activity can cause susceptibility to febrile seizures [21, 26, 27]. Specifically in the *sick* mutant flies, the inhibitory GABAergic neurons in the mushroom body may not reach their target neurons to inhibit their excitabilities properly [13, 42]. This may be one of the reasons why they are vulnerable to heat-induced seizures. Further reports of affected subjects will elucidate whether epilepsy may be part of the NAV2-related phenotypic spectrum.

Interestingly, the finding of congenital heart defects in the affected individual reported in this study may indicate a potential role of NAV2 also in the cytoskeleton dynamics of cardiac myocytes. This may be consistent with the observation of wide migration defects in the *Caenorhabditis elegans* unc-53 mutant, with abnormal developing myoblasts and excretory cells in addition to cells from the ventral nerve cord^45^. Notably, congenital anomalies beyond the CNS have not been assessed before in the hypomorphic Nav2 mouse model. However, additional (unidentified) genetic causes underlying the congenital heart defects in our patient cannot be excluded, as clinical ES would not cover intronic variants or complex genomic rearrangement.

## Conclusion

In conclusion, we report a novel neurodevelopmental disorder characterized by severe impairment of brain and cerebellar development and found its association with biallelic *loss-of-function* variants in *NAV2*. The neuroradiological phenotype of the affected individual is characterized by a complex brain malformation with a peculiar combination of cerebellar and brainstem malformations including vermian hypoplasia, extensive foliation defects, pontine hypo-dysplasia, and splayed thin superior cerebellar peduncles with a molar tooth-like configuration. Through functional analyses in human cells, the *Nav2* hypomorphic mouse, and the *NAV2* ortholog in flies (*sick*), our study highlights a potentially conserved role of NAV2 in regulating neuronal migration and CNS development across different species. The identification of other affected individuals carrying biallelic variants in *NAV2* will be fundamental to confirm the implication of this gene in the phenotype we observed and to characterize the clinical spectrum of the disease.

## Consortia

Members of The Undiagnosed Diseases Network (UDN) and SYNaPS are listed below.

## Members of the Undiagnosed Diseases Network

Mahshid S. Azamian^21^, Carlos A. Bacino^21^, Ashok Balasubramanyam^21^, Lindsay C. Burrage^21^, Hsiao-Tuan Chao^21^, Gary D. Clark^21^, William J. Craigen^21^, Hongzheng Dai^21^, Fariha Jamal^21^,

Lefkothea Karaviti^21^, Shamika Ketkar^21^, Brendan H. Lee^21^, Richard A. Lewis^21^, Ronit Marom^21^, Paolo M. Moretti^21^, Sarah K. Nicholas^21^, James P. Orengo^21^, Jennifer E. Posey^21^, Lorraine Potocki^21^, Daryl A. Scott^21^, Alyssa A. Tran^21^, Tiphanie P. Vogel^21^, Monika Weisz Hubshman^21^, Kim Worley^21^, Michael F. Wangler^21^, Shinya Yamamoto^21^, Christine M. Eng^21^, Pengfei Liu^21^, Patricia A. Ward^21^, Edward Behrens^22^, Kosuke Izumi^22^, Marni Falk^22^, Kelly Hassey^22^, Kathleen Sullivan^22^, Anna Raper^22^, Gonench Kilich^22^, Zhe Zhang^22^, Adeline Vanderver^22^, David B. Goldstein^23^, Heidi Cope^24^, Allyn McConkie-Rosell^24^, Kelly Schoch^24^, Vandana Shashi^24^, Edward C. Smith^24^, Rebecca C. Spillmann^24^, Jennifer A. Sullivan^24^, Queenie K.-G. Tan^24^, Nicole M. Walley^24^,

Pankaj B. Agrawal^25^, Alan H. ^25^, Gerard T. Berry^25^, LaurenC. Briere^25^, Laurel A. Cobban^25^, Matthew Coggins^25^, Cynthia M. Cooper^25^, Elizabeth L. Fieg^25^, Frances High^25^, Ingrid A. Holm^25^, Susan Korrick^25^, Joel B. Krier^25^, Sharyn A. Lincoln^25^, Joseph Loscalzo^25^, Richard L. Maas^25^, Calum A. MacRae^25^, J. Carl Pallais^25^, Deepak A. Rao^25^, Lance H. Rodan^25^, Edwin K. Silverman^25^, Joan M. Stoler^25^, David A. Sweetser^25^, Melissa Walker^25^, Chris A. Walsh^25^, Cecilia Esteves^25^, Isaac S. Kohane^25^, Kimberly LeBlanc^25^, Alexa T. McCray^25^, Shilpa N. Kobren^25^, Amelia L. M. Tan^25^, Rachel Mahoney^25^, Surendra Dasari^26^, Brendan C.Lanpher^26^, Ian R. Lanza^26^, Eva Morava^26^, Devin Oglesbee^26^, Guney Bademci^27^, Deborah Barbouth^27^, Stephanie Bivona^27^, Olveen Carrasquillo^27^, Ta Chen Pete Chang^27^, Irman Forghani^27^, Alana Grajewski^27^, Rosario Isasi^27^, Byron Lam, ^27^ Roy Levitt^27^, Xue Zhong Liu, Jacob McCauley^27^, Ralph Sacco^27^, Mario Saporta^27^, Judy Schaechter^27^, Mustafa Tekin^27^, Fred Telischi^27^, Willa Thorson^27^, Stephan Zuchner^27^, Heather A. Colley^28^, Jyoti G. Dayal^28^, David J. Eckstein^28^, Laurie C. Findley^28^, Donna M. Krasnewich^28^, Laura A. Mamounas^28^, Teri A. Manolio^28^, John J. Mulvihill^28^, Grace L. LaMoure^28^, Madison P. Goldrich^28^, Tiina K. Urv^28^, Argenia L. Doss^28^, Maria T. Acosta^28^, Carsten Bonnenmann^28^, Precilla D'Souza^28^, David D. Draper^28^, Carlos Ferreira^28^, Rena A. Godfreyv, Catherine A. Groden^28^, Ellen F. Macnamara^28^, Valerie V. Maduro, ^28^ Thomas C. Markello^28^, Avi Nath^28^, Donna Novacic^28^, Barbara N. Pusey^28^, Camilo Toro^28^, ColleenE. Wahl^28^, Eva Baker^28^, Elizabeth A. Burke^28^, David R. Adams, William A. Gahl^28^, May Christine V. Malicdan^28^, Cynthia J. Tifft^28^, Lynne A. Wolfe^28^, John Yang^28^, Bradley Power^28^, Bernadette Gochuico^28^, Laryssa Huryn^28^, Lea Latham^28^, Joie Davis^28^, Deborah Mosbrook-Davis^28^, Francis Rossignol^28^, Ben Solomon^28^, John MacDowall^28^, Audrey Thurm^28^, Wadih Zein^28^, Muhammad Yousef^28^, Margaret Adam^29^, Laura Amendola^29^, Michael Bamshad^29^, Anita Beck^29^, Jimmy Bennett^29^, Beverly Berg-Rood^29^, Elizabeth Blue^29^, Brenna Boyd^29^, PeterByers^29^, Sirisak Chanprasert^29^, Michae Cunningham^29^, Katrina Dipple^29^, Daniel Doherty^29^, Dawn Earl, Ian Glass^29^, Katie Golden-Grant^29^, Sihoun Hahn^29^, Anne Hing, Fuki M. Hisama^29^, Martha Horike-Pyne^29^, Gail P. Jarvik^29^, Jeffrey Jarvik^29^, Suman Jayadev^29^, Christina Lam^29^, Kenneth Maravilla^29^, Heather Mefford^29^, J. Lawrence Merritt^29^, Ghayda Mirzaa^29^, Deborah Nickerson^29^, Wendy Raskind^29^, Natalie Rosenwasser^29^, C. RonScott^29^, Angela Sun^29^, Virginia Sybert^29^, Stephanie Wallace^29^, Mark Wener^29^, Tara Wenger^29^, Euan A. Ashley^30^, Gill Bejerano^30^, Jonathan A. Bernstein^30^, Devon Bonner^30^, Terra R. Coakley^30^, Liliana Fernandez^30^, Paul G. Fisher^30^, Jason Hom^30^, Yong Huang^30^, Jennefer N.Kohler^30^, ElijahKravets^30^, Beth A. Martin^30^, Shruti Marwaha^30^, Archana N. Raja^30^, Chloe M. Reuter^30^, Maura Ruzhnikov^30^, Jacinda B. Sampson^30^, Kevin S. Smith^30^, Shirley Sutton^30^, Holly K. Tabor^30^, Brianna M Tucker^30^, Matthew T. Wheeler^30^, Diane B. Zastrow^30^, Chunli Zhao^30^, William E. Byrd^31^, Andrew B. Crouse^31^, Matthew Might^31^, Mariko Nakano-Okuno^31^, Jordan Whitlock^31^, Gabrielle Brown^32^, Manish J. Butte^32^, Esteban C. Dell'Angelica^32^, Naghmeh Dorrani^32^, Emilie D. Douine^32^, Brent L. Fogel^32^, Irma Gutierrez^32^, Alden Huang^32^, Deborah Krakow^32^, Hane Lee^32^, Sandra K. Loo^32^, Bryan C. Mak^32^, Martin G. Martin^32^, Julian A.Martínez-Agosto^32^, Elisabeth McGee^32^, Stanley F. Nelson^32^, Shirley Nieves-Rodriguez^32^, Christina G.S. Palmer^32^, Jeanette C. Papp^32^, Neil H. Parker^32^, Genecee Renteria^32^, Rebecca H. Signer^32^, Janet S. Sinsheimer^32^, Jijun Wan^32^, Lee-kai Wang^32^, Katherine Wesseling Perry^32^, Jeremy D. Woods32, Justin Alvey^33^, Ashley Andrews^33^, Jim Bale^33^, John Bohnsack^33^, Lorenzo Botto^33^, John Carey^33^, Laura Pace^33^, Nicola Longo^33^, Gabor Marth^33^, Paolo Moretti^33^, Aaron Quinlan^33^, Matt Velinder^33^, Dave Viskochil^33^, Pinar Bayrak-Toydemir^33^, Rong Mao^33^, Monte Westerfield^34^, Anna Bican^35^, Elly Brokamp^35^, Laura Duncan^35^, Rizwan Hamid^35^, Jennifer Kennedy^35^, Mary Kozuira^35^, John H. Newman^35^, John A. Phillips III^35^, Lynette Rives^35^, Amy K. Robertson^35^, Emily Solem^35^, Joy D.Cogan^35^, F. Sessions Cole^36^, Nichole Hayes^36^, Dana Kiley^36^, Kathy Sisco^36^, Jennifer Wambach^36^, Daniel Wegner^36^, Dustin Baldridge^36^, Stephen Pak^36^, Timothy Schedl^36^, Jimann Shin^36^, Lilianna Solnica-Krezel^36^.

## Study Group Members Affiliations – Undiagnosed Disease Network

21 Baylor College of Medicine, TX, TX 77,030, USA.

22 Children’s Hospital of Philadelphia, PA 19,104, USA.

23 Columbia University, NY, NY 10,027, USA.

24 Duke University, NC 27,708, USA.

25 Harvard University, Cambridge, MA, USA.

26 Mayo Clinic (Rochester, MN), USA.

27 University of Miami, FL 33,146, USA.

28 National Institutes of Health, MD 20,814, USA.

29 University of Washington and/or Seattle Children’s Hospital, WA 98,195, USA.

30 Stanford University, CA 94,305, USA.

31 University of Alabama Birmingham, AL 35,294, USA.

32 University of California, Los Angeles (UCLA), CA 90,095, USA.

33 University of Utah, UT 84,112, USA.

34 University of Oregon, OR 97,403, USA.

35 Vanderbilt University, TN 37,235, USA.

36 Washington University, St. Louis, MO 63,130, USA.

**The** **Syn** **aptopathies** **a** **nd** **P** **aroxysmal** **S** **yndromes (SYNaPS) Study Group (** http://neurogenetics.co.uk/synaptopathies-synaps/ **).**

## Study Group Members:

Stephanie Efthymiou^17^, Reza Maroofian^17^, Mhammed Aguennouz^19^, Yamna Kriouile^37^, Mohamed El Khorassani^38^, Blagovesta Karashova^38^, Daniela Avdjieva^38^, Hadil Kathom^38^, Radka Tincheva^38^, Lionel Van Maldergem^39^, Wolfgang Nachbauer^40^, Sylvia Boesch^40^, Larissa Arning^41^, Dagmar Timmann^42^, Vincenzo Leuzzi^43^, Belen Pérez-Dueñas^44^, Gabriella Di Rosa^45^, Erica Pironti^45^, Jatinder S. Goraya^46^, Tipu Sultan^47^, Salman Kirmani^48^, Shahnaz Ibrahim^49^, Farida Jan^49^, Jun Mine^50^, Selina Banu^51^, Pierangelo Veggiotti^52^, Michel D. Ferrari^53^, Arn M J M van den Maagdenberg^53^, Alberto Verrotti^54^, Gian Luigi Marseglia^55^, Salvatore Savasta^55^, Gian Vincenzo Zuccotti^56^, Carmela Scuderi^57^, Eugenia Borgione^57^, Valeria Dipasquale^58^, Maria Concetta Cutrupi^58^, Rosaria Nardello^59^, Benigno Monteagudo Sanchez^60^, Mercedes Pineda-Marfa’^61^, Francina Munell^61^, Alfons Macaya^61^, Richard Boles^62^, Gali Heimer^63^, Salvatore Leonardi^64^, Martino Ruggieri^64^, Rosalba De-Sarro^19^, Nancy Malintan^17^, Oscar D. Bello^17^, Maria Natalia Zanetti^17^, Henry Houlden^17^.

## Study Group Members Affiliations—SYNaPS

17 Department of Neuromuscular Diseases, UCL Institute of Neurology, London WC1N 3BG, UK.

37 Children's Hospital of Rabat, University of Rabat, Rabat 6527, Morocco.

38 Department of Paediatrics, Medical University of Sofia, Sofia 1431, Bulgaria.

39 Centre of Human Genetics, University Hospital Liege, Liege 4000, Belgium.

40 Department of Neurology, Medical University Innsbruck, Anichstrasse 35, Innsbruck 6020, Austria.

41 Department of Human Genetics, Ruhr-University Bochum, Bochum 44,801, Germany.

42 Braun Neurologische Universitätsklinik Universität Essen, Hufelandstr 55, Essen D-45122, Germany.

43 Department of Human Neuroscience, Unit of Child Neurology and Psychiatry, University La Sapienza, 00,185, Rome, Italy.

44 Hospital Sant Joan de Deu, Esplugues de Llobregat 08,950, Barcelona, Spain.

45 Department of Pediatrics, University of Messina, Messina 98,123, Italy.

46 Division of Paediatric Neurology, Dayanand Medical College & Hospital, Ludhiana, Punjab 141,001, India.

47 Department of Paediatric Neurology, Children's Hospital of Lahore, Lahore 381-D/2, Pakistan.

48 Department of Medical Genetics, Aga Khan University Hospital, Karachi, Karachi City, Sindh 74,800, Pakistan.

49 Department of Paediatric Neurology, Aga Khan University Hospital, Karachi, Karachi City, Sindh 74,800, Pakistan.

50 Department of Pediatrics, Shimane University School of Medicine, 89‐1 Enya, Izumo, Shimane 693‐8501, Japan.

51 Institute of Child Health and Shishu Shastho Foundation Hospital, Mirpur, Dhaka 1216, Bangladesh.

52 Pediatric Neurology Unit, V. Buzzi Children's Hospital, Via Castelvetro 32, 20,154 Milan, Italy.

53 Leiden University Medical Center, Albinusdreef 2, Leiden 2333, Netherlands.

54 Paediatric Department, San Salvatore Hospital, University of L'Aquila, L'Aquila, Italy.

55 Department of Pediatrics, University of Pavia, IRCCS Policlinico "San Matteo", Pavia 27,100, Italy.

56 Department of Biomedical and Clinical Sciences "L. Sacco", University of Milan; Paediatric Clinical Research Centre Fondazione "Romeo ed Enrica Invernizzi", University of Milano; Department of Paediatrics, Children's Hospital "V. Buzzi", Milano, Italy.

57 Laboratorio di Neuropatologia Clinica, U.O.S. Malattie, Neuromuscolari Associazione OASI Maria SS. ONLUS – IRCCS, Via Conte Ruggero 73, 94,018 Troina, Italy.

58 Department of Pediatrics, University Hospital “Gaetano Martino”, University of Messina, Messina 98,123, Italy.

59 Child Neuropsychiatry Unit, University of Palermo, Italy.

60 Hospital Arquitecto Marcide, Avenida de la Residencia S/N, Ferrol (A Coruña), 15,401 Spain.

61 Neuropediatrics Unit, University Hospital Vall d'Hebron, Barcelona 08,035, Spain.

62 Courtagen Life Sciences, 12 Gill Street Suite 3700, Woburn, MA 01,801 USA.

63 Division of Pediatric Neurology, Edmond and Lily Children's Hospital, Chaim Sheba Medical Center, 52,621 Ramat Gan, Israel.

64 Department of Clinical and Experimental Medicine, University of Catania, Catania, Italy.

## Supplementary Information

Below is the link to the electronic supplementary material.

Supplementary file1 (TIFF 197 KB)

Supplementary file2 (TIFF 1631 KB)

Supplementary file3 (DOCX 28 KB)

Supplementary file4 (PPTX 1403 KB)

Supplementary file5 (XLSX 19 KB)

Supplementary file6 (TIF 6487 KB)

Supplementary file7 (MP4 5117 KB)

Supplementary file8 (MP4 9747 KB)

## Data Availability

Additional information regarding the genetic and functional studies are available upon request to the corresponding author.
