# Supplementary figures and images for: Loss of Neuron Navigator 2 Impairs Brain and Cerebellar Development
Source: Cerebellum. 2022 Feb 26;22(2):206–22. doi: 10.1007/s12311-022-01379-3 (PMC9985553; doi:10.1007/s12311-022-01379-3)

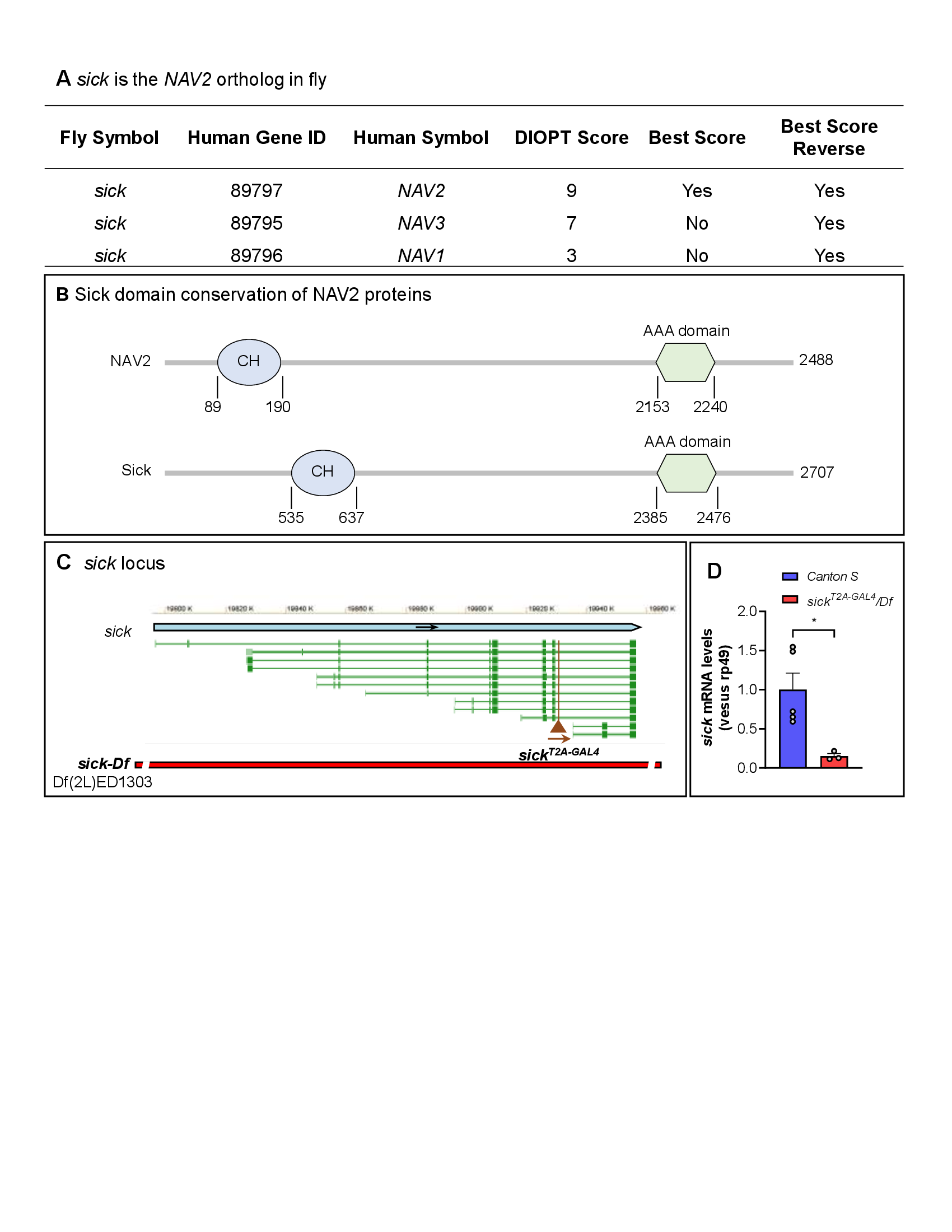

Supplement: Supplementary file 1 — Supplementary file1 (TIFF 197 KB) [file 12311_2022_1379_MOESM1_ESM.tiff]

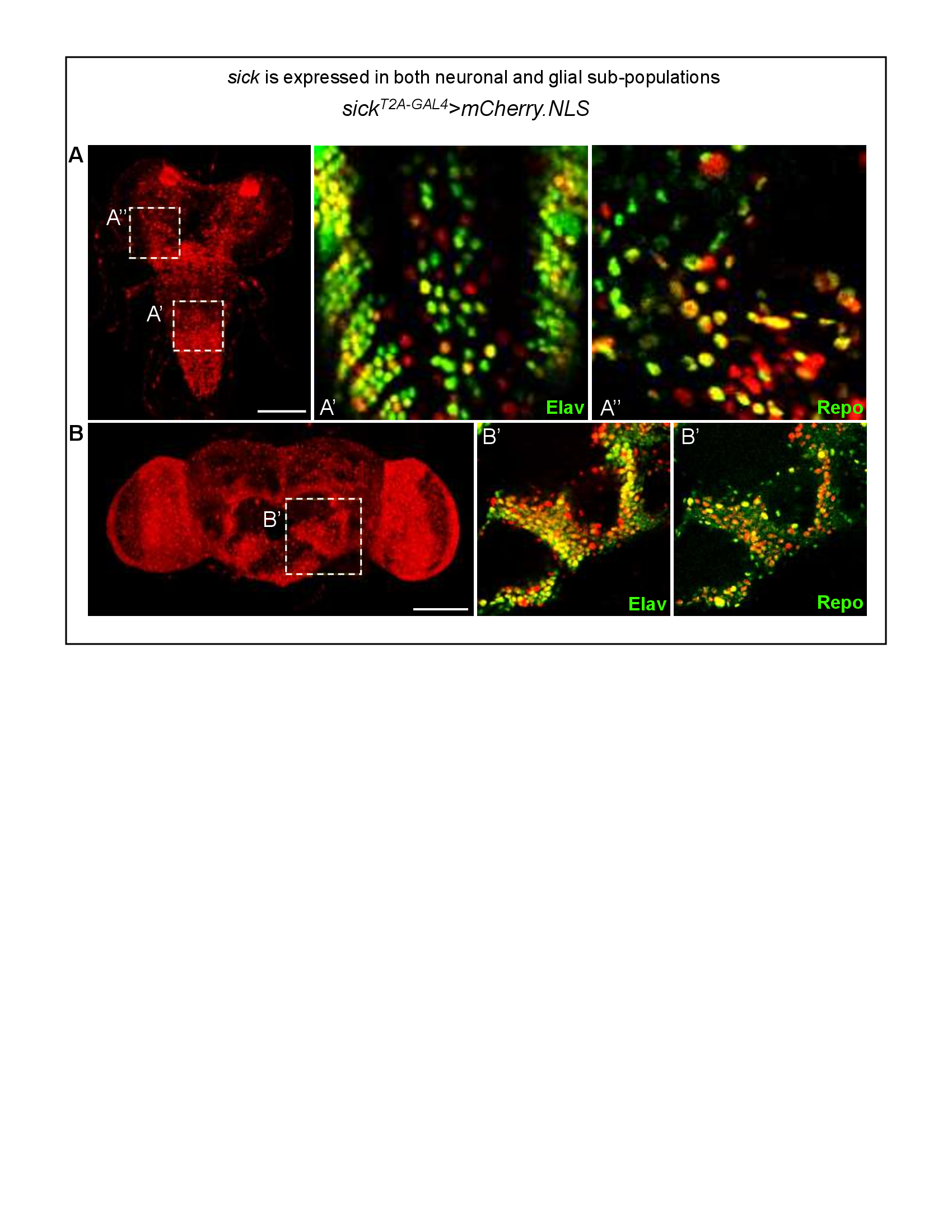

Supplement: Supplementary file 2 — Supplementary file2 (TIFF 1631 KB) [file 12311_2022_1379_MOESM2_ESM.tiff]

## Slide 1
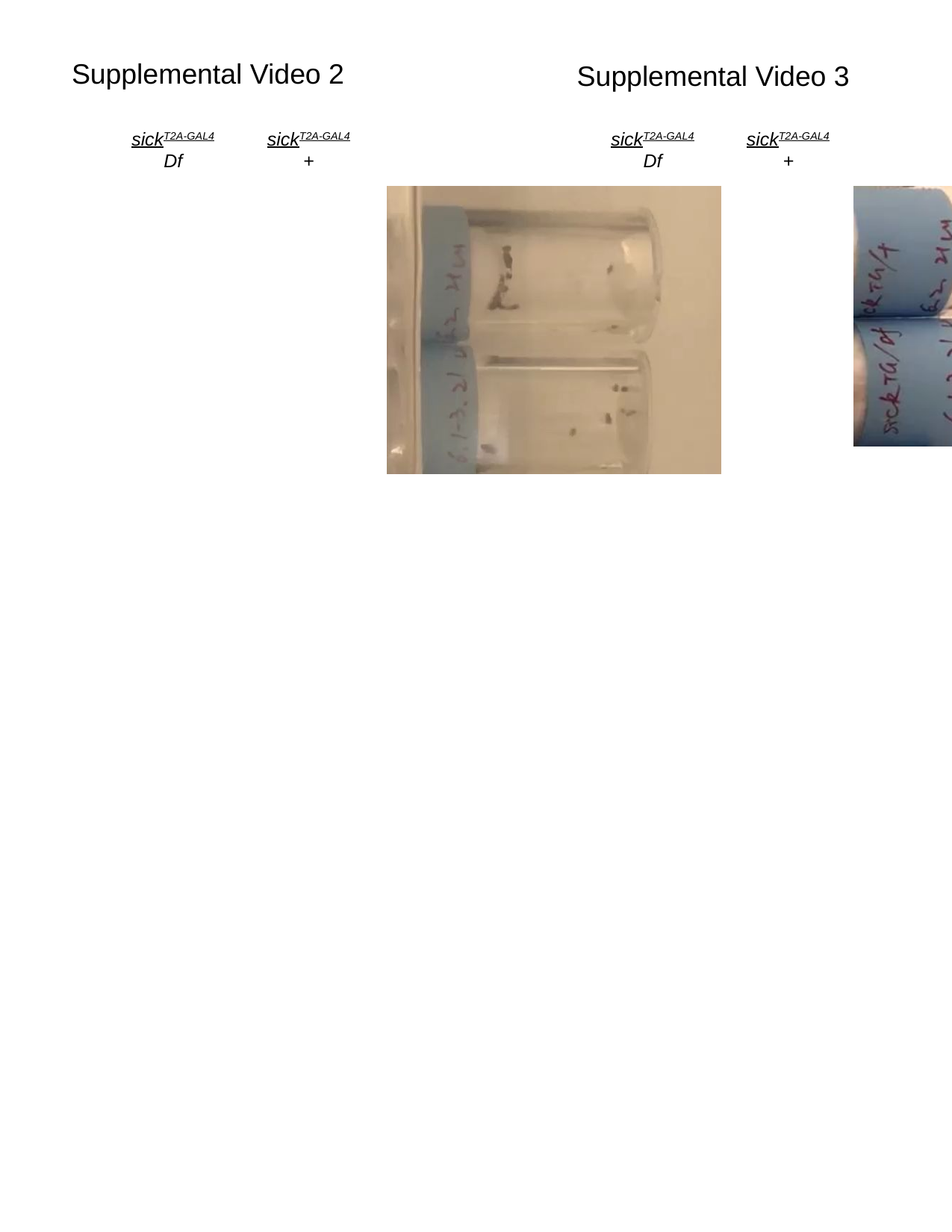

Supplemental Video 2
Supplemental Video 3
sickT2A-GAL4
Df
sickT2A-GAL4
+
sickT2A-GAL4
Df
sickT2A-GAL4
+

Supplement: Supplementary file 4 — Supplementary file4 (PPTX 1403 KB) [file 12311_2022_1379_MOESM4_ESM.pptx]

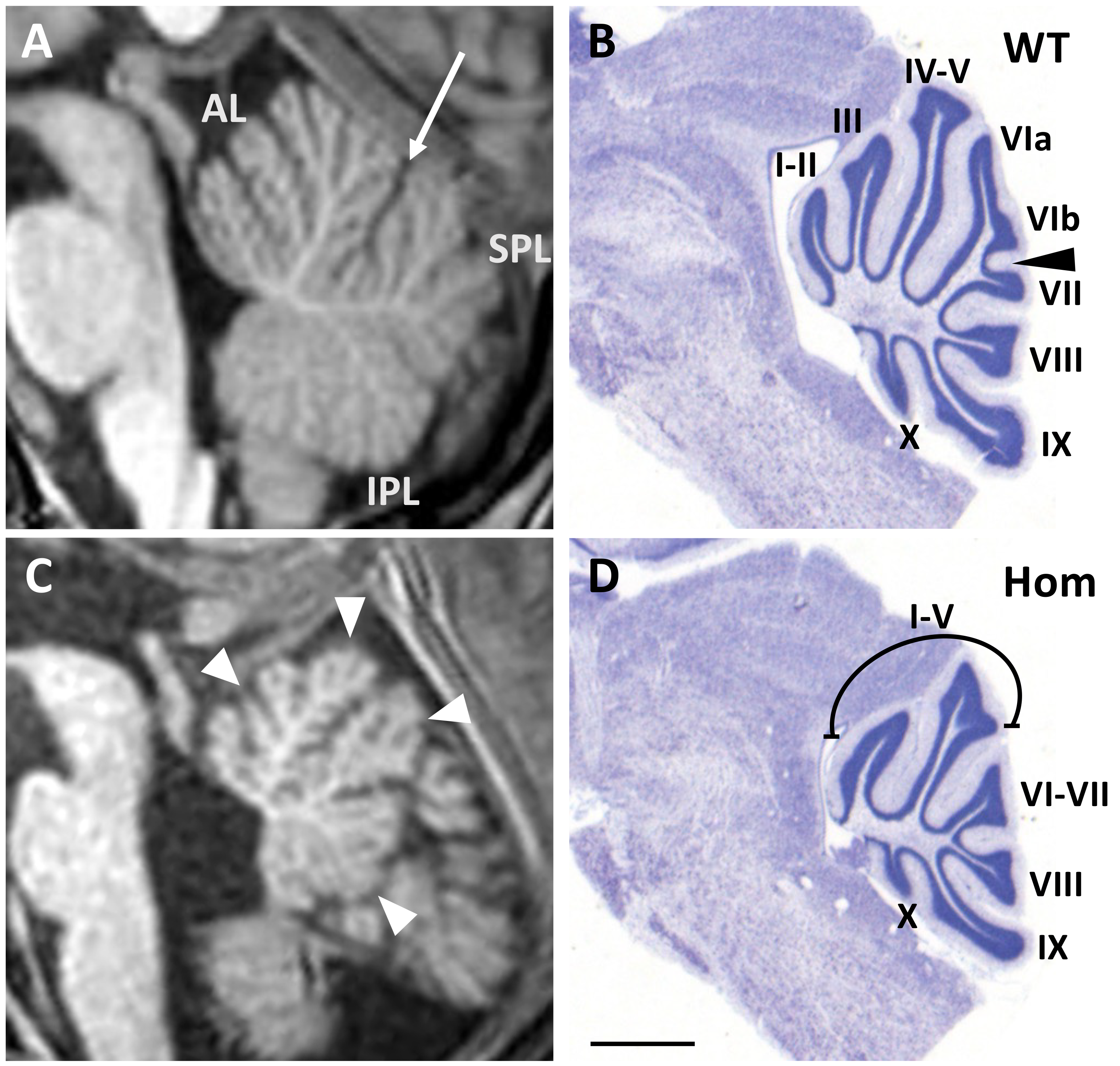

Supplement: Supplementary file 6 — Supplementary file6 (TIF 6487 KB) [file 12311_2022_1379_MOESM6_ESM.tif]
