# Supplementary material for: Loss of Neuron Navigator 2 Impairs Brain and Cerebellar Development
Source: Cerebellum. 2022 Feb 26;22(2):206–22. doi: 10.1007/s12311-022-01379-3 (PMC9985553; doi:10.1007/s12311-022-01379-3)
Supplement: Supplementary file 3 — Supplementary file3 (DOCX 28 KB) [file 12311_2022_1379_MOESM3_ESM.docx]

**Supplemental material**

**Supplemental Table 1. Loss of function variants reported on the gnomAD database**

**Supplemental Figure 1. Comparison of cerebellar vermis features in the patient with NAV2 deficiency versus the *Nav2*  (*unc-53H2*) hypomorphic mutant mouse**

A) Sagittal reconstructed 3D T1-weighted image of a control subject revealing normal vermis anatomy. Note the presence of the primary fissure (arrow). AL: anterior lobe; SPL: superior posterior lobe; IPL: inferior posterior lobe.  B) Medial Nissl-stained sagittal section (30 μm) showing normal cerebellar vermis foliation in the wild-type mouse. The intercrural fissure separating VII and VIII is show by the arrowhead. C) Sagittal reconstructed 3D T1-weighted image of the affected subject reveals marked vermal hypoplasia and dysplasia (arrowheads) with prevalent involvement of the anterior and superior posterior lobe. Note the mild upward rotation of the vermis. D) Abnormal foliation in the Nav2 hypomorphic mutant mouse. Note the presence of only two folia in the region of I-V compared to three distinct folia in the wild-type, and underdevelopment of IV/VII with absence of the intercrural fissure.

**Supplemental Figure 2. Sick is the NAV2 ortholog in fly**

A) Prediction of Sick homologs in human with DIOPT scores. Data were obtained from DIOPT (DRSC Integrative Ortholog Prediction Tool). B) NAV2 and Sick domain structures. C) Genomic structure of the *Drosophila* *sick* locus and reagents used in this study. D) Bar graph showing sick mRNA quantification by real time PCR with. Each dot contains 3~5 adult flies. Data are represented as mean ± SEM. Unpaired t test, *P < 0.05.

**Supplemental Figure 3. Sick is expressed in both neuronal and glial sub-populations**

Gene expression of sick by crossing *sick^T2A-GAL4^* to *UAS-mCherry.NLS* and examining third instar larva (A) or adult brain (D) by confocal microscopy. Co-localization with neuronal marker (Elav, green color) and glial marker (Repo, green color) were shown in single-slice images. Dashed squares indicate regions used in A’, A’’ and B’. When both nuclear markers colocalize with each other, they are yellow. Scale bars = 100 µm.

**Videos**

Video 1 showing broad based gait and video 2 showing impairment of voluntary saccadic eye movements.

Video 3: *sick* mutants show rapid onset of seizure like behaviour when submerged into 42 °C water.

Video 4: The recovery of *sick* mutants from heat induced seizures.

**Members of the Undiagnosed Diseases Network**

Maria T. Acosta

Margaret Adam

David R. Adams

Pankaj B. Agrawal

Mercedes E. Alejandro

Justin Alvey

Laura Amendola

Ashley Andrews

Euan A. Ashley

Mahshid S. Azamian

Carlos A. Bacino

Guney Bademci

Eva Baker

Ashok Balasubramanyam

Dustin Baldridge

Jim Bale

Michael Bamshad

Deborah Barbouth

Pinar Bayrak-Toydemir

Anita Beck

Alan H. Beggs

Edward Behrens

Gill Bejerano

Jimmy Bennet

Beverly Berg-Rood

Jonathan A. Bernstein

Gerard T. Berry

Anna Bican

Stephanie Bivona

Elizabeth Blue

John Bohnsack

Carsten Bonnenmann

Devon Bonner

Lorenzo Botto

Brenna Boyd

Lauren C. Briere

Elly Brokamp

Gabrielle Brown

Elizabeth A. Burke

Lindsay C. Burrage

Manish J. Butte

Peter Byers

William E. Byrd

John Carey

Olveen Carrasquillo

Ta Chen Peter Chang

Sirisak Chanprasert

Hsiao-Tuan Chao

Gary D. Clark

Terra R. Coakley

Laurel A. Cobban

Joy D. Cogan

Matthew Coggins

F. Sessions Cole

Heather A. Colley

Cynthia M. Cooper

Heidi Cope

William J. Craigen

Andrew B. Crouse

Michael Cunningham

Precilla D'Souza

Hongzheng Dai

Surendra Dasari

Joie Davis

Jyoti G. Dayal

Matthew Deardorff

Esteban C. Dell'Angelica

Shweta U. Dhar

Katrina Dipple

Daniel Doherty

Naghmeh Dorrani

Argenia L. Doss

Emilie D. Douine

David D. Draper

Laura Duncan

Dawn Earl

David J. Eckstein

Lisa T. Emrick

Christine M. Eng

Cecilia Esteves

Marni Falk

Liliana Fernandez

Carlos Ferreira

Elizabeth L. Fieg

Laurie C. Findley

Paul G. Fisher

Brent L. Fogel

Irman Forghani

William A. Gahl

Ian Glass

Bernadette Gochuico

Rena A. Godfrey

Katie Golden-Grant

Alica M. Goldman

Madison P. Goldrich

David B. Goldstein

Alana Grajewski

Catherine A. Groden

Irma Gutierrez

Sihoun Hahn

Rizwan Hamid

Neil A. Hanchard

Athena Hantzaridis

Kelly Hassey

Nichole Hayes

Frances High

Anne Hing

Fuki M. Hisama

Ingrid A. Holm

Jason Hom

Martha Horike-Pyne

Alden Huang

Yong Huang

Laryssa Huryn

Rosario Isasi

Fariha Jamal

Gail P. Jarvik

Jeffrey Jarvik

Suman Jayadev

Lefkothea Karaviti

Jennifer Kennedy

Dana Kiley

Shilpa N. Kobren

Isaac S. Kohane

Jennefer N. Kohler

Deborah Krakow

Donna M. Krasnewich

Elijah Kravets

Susan Korrick

Mary Koziura

Joel B. Krier

Seema R. Lalani

Byron Lam

Christina Lam

Grace L. LaMoure

Brendan C. Lanpher

Ian R. Lanza

Lea Latham

Kimberly LeBlanc

Brendan H. Lee

Hane Lee

Roy Levitt

Richard A. Lewis

Sharyn A. Lincoln

Pengfei Liu

Xue Zhong Liu

Nicola Longo

Sandra K. Loo

Joseph Loscalzo

Richard L. Maas

John MacDowall

Ellen F. Macnamara

Calum A. MacRae

Valerie V. Maduro

Bryan C. Mak

May Christine V. Malicdan

Laura A. Mamounas

Teri A. Manolio

Rong Mao

Kenneth Maravilla

Thomas C. Markello

Ronit Marom

Gabor Marth

Beth A. Martin

Martin G. Martin

Julian A. Martínez-Agosto

Shruti Marwaha

Jacob McCauley

Allyn McConkie-Rosell

Alexa T. McCray

Elisabeth McGee

Heather Mefford

J. Lawrence Merritt

Matthew Might

Ghayda Mirzaa

Eva Morava

Paolo M. Moretti

Deborah Mosbrook-Davis

John J. Mulvihill

David R. Murdock

Anna Nagy

Mariko Nakano-Okuno

Avi Nath

Stan F. Nelson

John H. Newman

Sarah K. Nicholas

Deborah Nickerson

Shirley Nieves-Rodriguez

Donna Novacic

Devin Oglesbee

James P. Orengo

Laura Pace

Stephen Pak

J. Carl Pallais

Christina GS. Palmer

Jeanette C. Papp

Neil H. Parker

John A. Phillips III

Jennifer E. Posey

Lorraine Potocki

Bradley Power

Barbara N. Pusey

Aaron Quinlan

Wendy Raskind

Archana N. Raja

Deepak A. Rao

Genecee Renteria

Chloe M. Reuter

Lynette Rives

Amy K. Robertson

Lance H. Rodan

Jill A. Rosenfeld

Natalie Rosenwasser

Francis Rossignol

Maura Ruzhnikov

Ralph Sacco

Jacinda B. Sampson

Susan L. Samson

Mario Saporta

C. Ron Scott

Judy Schaechter

Timothy Schedl

Kelly Schoch

Daryl A. Scott

Vandana Shashi

Jimann Shin

Rebecca Signer

Edwin K. Silverman

Janet S. Sinsheimer

Kathy Sisco

Edward C. Smith

Kevin S. Smith

Emily Solem

Lilianna Solnica-Krezel

Ben Solomon

Rebecca C. Spillmann

Joan M. Stoler

Jennifer A. Sullivan

Kathleen Sullivan

Angela Sun

Shirley Sutton

David A. Sweetser

Virginia Sybert

Holly K. Tabor

Amelia L. M. Tan

Queenie K.-G. Tan

Mustafa Tekin

Fred Telischi

Willa Thorson

Audrey Thurm

Cynthia J. Tifft

Camilo Toro

Alyssa A. Tran

Brianna M. Tucker

Tiina K. Urv

Adeline Vanderver

Matt Velinder

Dave Viskochil

Tiphanie P. Vogel

Colleen E. Wahl

Stephanie Wallace

Nicole M. Walley

Chris A. Walsh

Melissa Walker

Jennifer Wambach

Jijun Wan

Lee-kai Wang

Michael F. Wangler

Patricia A. Ward

Daniel Wegner

Mark Wener

Tara Wenger

Katherine Wesseling Perry

Monte Westerfield

Matthew T. Wheeler

Jordan Whitlock

Lynne A. Wolfe

Jeremy D. Woods

Shinya Yamamoto

John Yang

Muhammad Yousef

Diane B. Zastrow

Wadih Zein

Chunli Zhao

Stephan Zuchner
